# Identification of a potential interspecies reassortant rotavirus G and avastrovirus 2 co-infection from black-headed gull (*Chroicocephalus ridibundus*) in Hungary

**DOI:** 10.1371/journal.pone.0317400

**Published:** 2025-03-24

**Authors:** Péter Pankovics, Károly Takáts, Péter Urbán, Róbert Mátics, Gábor Reuter, Ákos Boros

**Affiliations:** 1 Department of Medical Microbiology and Immunology, Medical School, University of Pécs, Pécs, Hungary; 2 János Szentágothai Research Centre of the University of Pécs, Bioinformatics Research Group, Genomics and Bioinformatics Core Facility, Pécs, Hungary; 3 Hungarian Nature Research Society, Ajka, Hungary; 4 Department of Behavioural Science, Medical School, University of Pécs, Pécs, Hungary; Federal Medical Centre Abeokuta, NIGERIA

## Abstract

The black-headed gull is the most common nesting gull species in Hungary. Based on the lifestyle and feeding habits of the black-headed gull, which is highly adapted to the human environment, they can be carriers and spreaders of potential human and other animal pathogens. Between 2014 and 2018 within the framework of the “Life Bird Ringing program” a total of 7 faecal samples were collected from gulls and one sample (MR04) was randomly selected for viral metagenomics and mass sequencing. 95.4% and 4% of the reads were classified into family *Seadornaviridae* and *Astroviridae*, respectively, and then were verified by RT-PCR method. In this study, the complete genome of a potential interspecies reassortant rotavirus (RV) strain gull/MR04_RV/HUN/2014 (PP239049-PP239059) and the partial ORF1ab, complete ORF2 of a novel avian nephritis virus strain gull/MR04_AAstV/HUN/2014 (PP239060) was discussed. The strain gull/MR04_RV/HUN/2014 was closely related to rotavirus G (RVG) viruses based on the proteins VP1–VP3, VP6, NSP2, NSP3, and NSP5, but it was more related to the human rotavirus B (RVB) strain Bang373 based on the NSP1, NSP4 and VP7, VP4 proteins, which is assumed to be the result of reassortment between different RVG-RVB rotavirus species. The strain gull/MR04_AAstV/HUN/2014 belonged to the genus *Avastrovirus* species avastrovirus 2 (AAstV-2) and is related to members of group 6 of avian nephritis viruses (ANVs), but based on the genetic distances it may be the first representative of a separate group. Additional gull samples were found to be negative by RT-PCR. Gulls, which are well adapted to the human environment, could potentially spread enterically transmitted viral pathogens like interspecies reassortant rotaviruses (RVG/RVB), but further molecular surveillance is needed to explore more deeply the viral communities of gulls or other related species adapted to human environments.

## Introduction

Gulls belong to the family *Laridae* of birds (Aves), which are a group well adapted to the human environment found worldwide. The species of common black-headed gull (*Chroicocephalus ridibundus*) is the most common nesting gull species in Hungary (https://mme.hu/magyarorszagmadarai/madaradatbazis-larrid) and widespread species in Europe, too (http://datazone.birdlife.org/species/factsheet/black-headed-gull-larus-ridibundus) [1]. In terms of its lifestyle, it is a short-term migratory bird species, its domestic breeding population is 4,000–6,400 pairs (2015–2017) in Hungary, and its wintering area is the central and western part of the Mediterranean, and Western Europe. Despite its frequency, it is a protected species (least concern/LC) in Hungary. Regarding its diet, the species exhibits a highly adaptable feeding behaviour, provisioning its young with the most accessible food sources. In agricultural landscapes, it forages for insects, amphibians, invertebrates, and small mammals unearthed by fresh ploughing. Urban areas and their surroundings, however, offer a more abundant and easily accessible variety of resources, including waste from garbage dumps, carrion sites, and fish markets. This species may act as a carrier and vector for pathogens that pose risks to humans and other animals. To better understand these dynamics, next-generation sequencing (NGS) methods could be employed to identify and characterize potential pathogens, providing valuable insights into their transmission and impact [2].

Rotaviruses (RVs) (family *Seadornaviridae*) and astroviruses (family *Astroviridae*) are one of the most important and most frequently occurring viral pathogens in faeces which have the important pathogenic potential for humans and livestock (porcine/bovine RVs; porcine mamastro-, and chicken/turkey avastroviruses) [3,4]. The RV genome consists of 11 dsRNA segments that encode eight nonstructural proteins (RdRp, Cap and NSP1–NSP5/6), and six structural proteins (VP1–VP4, VP6, and VP7). Based on the analysis of conservative genome segments (e.g., Seg1) comparisons [5] and phylogenetic analysis of the intermediate capsid protein (VP6) (Seg6) [6], RVs can currently be classified into 9 groups A to J recognized by the International Committee on Taxonomy of Viruses (ICTV), where rotavirus A (RVA), C (RVC), D (RVD) and F (RVF) forming RVA-like clade (clade 1) and rotavirus B (RVB), G (RVG), H (RVH), I (RVI), J (RVJ) and forming RVB-like clade (clade 2) [3,5,7,8]. Additional strains are awaiting taxonomic classification representing novel RVL and RVK groups [9]. Species in RVA, RVD, RVF and RVG have been identified in bird species [8–10]. Several evolutionary mechanisms (mutation, recombination, and reassortment) have already been documented between strains of the same RV species [11–17], and recombination events between different species (interspecies) were also reported [18,19], but no information is available on reassortment between segments of different RV species.

Astroviruses have a positive sense, single-stranded RNA genome, having a genome-linked viral protein (VPg) on the 5’ terminal side and a polyadenylated tail (poly(A)) on the 3’ end. Astroviruses were initially considered as an etiological agent of enteric disease [20]; however, astroviruses have already been identified from infections causing central nervous system disease [21], hepatitis [22] and interstitial nephritis with growth retardation [23] in many mammalian and bird species. It seems that astroviruses have a broad host range and genetic diversity [24]. Currently, the members of the genus *Mammastrovirus* are infecting mammals and the members of the genus *Avastrovirus* could infect birds were distinguished [4,24]. Currently, species classification is based on comparative genetic analyses of the ORF2 capsid protein transcribed from sgRNA [25].

In this study, a faecal sample of a common black-headed gull was investigated by the NGS method and data was analyzed by an “in-house” data processing pipeline. We report the detection and complete genome characterization of a novel RV and an avastrovirus (AAstV) from a common black-headed gull in Hungary.

## Materials and methods

### Sample collection

A total of seven faecal samples were collected as part of the “Life Bird Ringing” program between 2014 and 2018 in Rétszilas, Hungary [26]. These included five samples from common black-headed gulls (*Chroicocephalus ridibundus*) (HA15097, MR03, MR04, MR05, and SH05705) and two samples from yellow-legged gulls (*Larus michahellis*) (RE00320 and RE00999). In Hungary, the annual number of ringed black-headed gulls typically ranges from a few hundred individuals, with the highest recorded number being 2,246. The breeding population sampled in Rétszilas consisted of 80–100 breeding pairs. In contrast, the yellow-legged gull has a significantly smaller population size, with fewer than 10 breeding pairs, a trend that is observed nationwide and is not limited to Rétszilas. The licensed ringer and their assistants entered the nesting colony for 3–4 hours on a suitable day in late May or early June, depending on the age of the chicks, to apply leg bands and collect faecal samples during defecation events. Approximately 2–5 grams of faecal samples were collected as a one-off from gull chicks on the nesting island during the ringing process. The samples were transferred into Eppendorf tubes and stored at −20°C for subsequent analysis.

### Ethics statement

The black-headed gull (*Chroicocephalus ridibundus*) is listed as “Least Concern” by both eBird (https://ebird.org/), the International Union for Conservation of Nature’s Society of the Red List of Threatened Species (https://www.iucnredlist.org/) and the BirdLife International (https://www.birdlife.org/). The Hungarian Ornithological and Nature Association (MME) (https://mme.hu/) works internationally as a member of the BirdLife International Partnership and undertakes practical work (survey, observation and ringing) with valid permission to conserve Hungary’s biodiversity. During the “Life bird ringing” program, the faecal samples were collected non-invasively by qualified ornithologists with valid permission (14/3859-9/2012, PE-KTF/97-13/2017), preserving the physical fitness of the birds. The ornithologist’s currently valid certificate (TMF-1180/22/2003) was issued by the National Inspectorate for Environment, Nature and Water.

### Sample preparation for viral metagenomics and next-generation sequencing

The faecal samples were homogenized mechanically with 0.1 M phosphate-buffered saline (35–40V/v%) and the total RNA was extracted with TRI Reagent (MRC, USA) according to the manufacturer’s protocol. 200 µL supernatant of faecal suspension of sample MR04 selected for viral metagenomics and next-generation sequencing (VM-NGS) analyses as described previously [27,28]. Briefly, the sample was first filtered through a 0.45 µm filter (Millipore), treated with Nuclease-mix (Turbo DNase, Ambion; Baseline-Zero. Epicentre; Benzonase, Novagen and RNase Promega). After total nucleic acid isolation (Quick-RNA Viral Kit, Zymo Research, Irvine, USA) and reverse transcription, DNA was amplified by random PCR. cDNA library was constructed by NEBNext Ultra II FS DNA Library Prep Kit for Illumina (NEB, Ipswitch, MA, USA). Illumina sequencing was performed on NovaSeq 6000 instrument (Illumina, San Diego, CA, USA) with a 2x151 run configuration [27,28]. The generated NGS sequence data was filtered, analyzed and classified using an in-house assembled bioinformatics pipeline which includes the Kaiju and Diamond algorithms [28]. Virus-related reads were verified using Megan and NCBI BlastX methods. Detailed bash and python scripts and settings used in this study for data filtering, creation and management of the Kaiju database, and Diamond-based validation are available upon request.

### Sample preparation for RT-PCR “screening” and Sanger sequencing

RV and astrovirus-related reads were aligned (MUSCLE aligner, https://www.ebi.ac.uk/jdispatcher/msa/muscle) to the most closely related sequences found in the GenBank (by BLAST searches). The alignments were visualized using GeneDoc and Geneious Prime software. Based on the aligned reads, several read-specific primers were designed to characterize the full-length viral sequences using conventional RT-PCR reactions, primer-walking [29], terminal deoxyribonucleotide transferase-based [30] and adapter-ligation-based 5′/3′ RACE [31,32] techniques. Based on multiple sequence alignments of reference and study strains, generic primer-pairs for VP6 of RV (MR04-GVP6-SCR-R: 5′-TGACACCAWACYTTRTC-3′, MR04-GVP6-SCR-F: 5′-ATCAATGATTACAATGC-3′, W: A/T, Y: C/T, R: A/G) and sequence-specific primer-pair for AAstV (MR04-AvAst2-SCR-R: 5′-GTTCATTACCTCAGACACATG-3′, MR04-AvAst2-F: 5′-AAGAGCTGGATGGTGGCTT-3′) were designed for epidemiological investigations. PCR products were directly sequenced with BigDye Terminator v1.1 (Thermo-Fisher) and run on an automated Sanger sequencer (Genetic Analyzer 3500, Applied Biosystems, Hitachi, Japan). Oligonucleotides were ordered from Integrated DNA Technologies (IDT DNA, https://eu.idtdna.com).

### Comparative and phylogenetic analysis of sequence data

Differences between nucleotide sequences were determined using Needleman-Wunsch global alignment (https://blast.ncbi.nlm.nih.gov/Blast.cgi), where identities (%), gaps (%) and NW Score were used for analysis and the calculated values were visualized by R/RStudio. Genetic clustering measurements were estimated using with R/RStudio with the help of “seqinr”, “msa”, “cluster”, “factoextra”, “randomcoloR”, “ggrepel” and “ape” libraries. Scripts for graphing NW data and cluster analysis of sequences are available on request. The nucleotide sequences of segments 6 and 11 were aligned by Multiple alignment program based on fast Fourier transform (MAFFT) v7.525 (2024/Mar/13; https://www.ebi.ac.uk/jdispatcher/msa/mafft) [33] and then analysed using IQ-TREE software using the following parameters: -m MFP, -alrt 1000, -T AUTO, -bb 100 [34–36]. According to the Bayesian Information Criterion (BIC) score the best-fit model for the VP6 dataset was TIM2 + F + R3 and for the NSP5 dataset was TIM2 + F + I + G4 models, respectively. Both the SH-aLRT test and the ultrafast bootstrap (UFBoot) values were set up to 1000 is each tree creation, and then the maximum likelihood consensus tree was used. The similarity analysis between the study strain gull/MR04-RV/HUN/2014 (RVG) and the ruddy turnstone rotavirus isolate MW05 (RVG) and human rotavirus B strain Bang373 (RVB) sequences included in the analysis was made using SimPlot 3.5.1 software [37]. For the analysis, the entire genomes of the study strain gull/MR04-RV/HUN/2014 genome segments (PP239049–PP239059) rotavirus, the closest relative ruddy turnstone rotavirus isolate MW05 (MH453863–MH453873) and human rotavirus B strain Bang373 (EU490415, EU490418, AY238384, AY238385, AY238388–AY238394) were concatenated and aligned using MAFFT method. The aligned nucleotide sequences were analysed with the SimPlot program based on the following setup: Window: 500 bp, Step: 20 bp, GapStrip: On, F84 (“Maximum Likelihood”), T/t: 2,0). Phylogenetic analysis of additional segments of RV, the deduced amino acid sequences were aligned using the MEGA11/Muscle method with default settings and the aligned amino acid sequences were tested with MEGA11/Find Best DNA/protein model search [38]. The statistical method determined by the lowest BIC scores was chosen as the basis for the phylogenetic analysis in each tree (S3 Fig). The phylogenetic analysis of astroviruses capsid sequences was aligned by the ClustalW method in MEGA11 and MAFFT alignment method, respectively (S4 Fig) and was tested with MEGA11/Find Best DNA/protein model search [38]. Maximum Likelihood statistical method, Lg with freqs (+F) model was chosen to estimate phylogenetic analysis. Rates among sites were calculated with Gamma Distributed with invariant sites (G + I) option and bootstrap (1000 replicates) was also used. After the pre-defined groups were formed, the mean group distances within groups were calculated with MEGA11 software [38]. The cluster analysis was performed using a script written in R (available for publication upon request) on avian nephritis viruses (ANV) sequences belonging to group 6.

### Placement of sequences in a public database

The complete nucleotide sequences of all genomic segments of RV strain gull/MR04_RV/HUN/2014 (PP239049-PP239059) and the partial ORF1ab-complete ORF2 sequence of AAstV strain gull/MR04_AAstV/HUN/2014 (PP239060) were submitted to the GenBank database in National Library of Medicine (NIH) also shared with DNA DataBank of Japan (DDBJ) and the European Nucleotide Archive (ENA).

## Results

After the bioinformatic analysis, a total of 1,305,096 virus-related reads were obtained which can be classified into virus families infecting protozoa (N = 504), bacteria (N = 24), plants (N = 18,108), fungi (N = 194), eucaryotic algae (N = 36), invertebrates (N = 220) and vertebrates (N = 1,286,010) (S1 Fig). The viral families infecting vertebrates (98.5% of the virus-related reads) were selected for further analysis where the reads can be potentially classified into family *Sedoreoviridae* (95.4%)*, Astroviridae* (4%), less than 1% was the sum of families *Coronaviridae, Picobirnaviridae, Caliciviridae, Retroviridae* and *Picornaviridae* (S1 Fig.).

### Characterization of RV strain gull/MR04_RV/HUN/2014

The determined complete genome of RV strain gull/MR04_RV/HUN/2014 (PP239049-PP239059) is 18,352 nucleotide (nt) long. The lengths and detailed descriptions of the segments were found in the S1 Table. The 5′ and 3′ non-coding regions (NCRs) of each segment have a length of 9 to 209 nt in the 5′ NCR and 30 to 112 nt in the 3′ NCR (S1 Table). At the extreme termini of each 5′/3′ NCRs of gull/MR04_RV/HUN/2014, conserved nucleotide sequence motifs were identified and analysed compared to the corresponding region of the reference strains. The common nucleotide sequences were seen in S2 Fig.

The amino acid (aa) sequences encoded by the gull/MR04_RV/HUN/2014 genome segments were compared with the corresponding homologues of the reference RV sequences belonging to groups A to D and F to J. The conservative motifs and “hotspots” analysed by Trojnar et al. [8] were looked for and aas that have changed compared to the group A RV isolate RVA/Simian-tc/ZAF/SA11-H96/1958/G3P5B[2] sequence in bold.

In the VP1, VP3 and NSP2, NSP3 proteins several conserved aa motifs could be detected with some aa changes. Of the six aa motifs described for RV VP1 protein, the **motif A** (YTD*VS*Q*WD*ASQHNT), the **motif B** (RYHGVAS*G*E*K*TTK*IGNSYANIALITTV), the **motif C** (THLRVDGD*D*NVVTAYTS), the **motif D** (MNARVKALASYTGLEMAKR), the **motif E** (KALASYTGLEMAK*RFIICGKIFERGA) and **motif F** (YPLDRLEAYSILPWP) could be found with minor/major changes and aa residues in motifs with a key role in catalysis are marked with an asterisk [8,39,40]. No aa residues interacting with the γ-phosphate of the incoming NTP and nucleosides in the P or N site could be found in motif F [39], but additional conservative motifs could be observed (AKxxSxPMx, xSxxFHVG, x represent any aa) at the N-terminal site of the protein.

After aligning the VP3 protein sequences, neither the KxTAMD nor the Kx[P/N]G motifs were found in the viral protein of the study strain [8], but aa motifs: GxxxE(S/T → GPEFES and LΩxL(S/T)NxxN (where Ω indicates an aromatic aa) → LYSISNTKN in the N7-MTase domain, aa motifs: SGHΦ (where Φ is a hydrophobic residue) → SGHL in the GTPase/RTPase domain and two HΦ(T/S)Φ motifs: HITL and HMTI in the VP3 C terminus (a predicted structural domain described in RVA) have been observed [41]. Further aa residues have also been detected in VP3 (one residue in N7-MTase domain: VRETVF → VRNTVF and four residues in the GTPase/RTPase domain: SRRSQFLR → RQVSIYNR; PMRWAK → PMRWSK and DRHSLH → EKHTRE) [41].

In the NSP2 protein, the conserved aa motif of the NTPase domain (KITxNxxxxxxDxxxxxxxAxxxxxxNxFAxIxHGxxHxR) was observed based on the structural similarity in RVA, RVC and RVD strains [8], of which the histidine triad (HIT motif: HΦHΦHΦΦ, Φ is a hydrophobic residue) were the conserved residues [40,42]. The modified HIT-like motif (**H**G**H**G**H**VRSV, catalytically active histidine bolded) was also detected in the study strain gull/MR04_RV/HUN/2014 NSP2.

Based on the NCBI Conserved Domain Search (CDS) the NSP3 protein of the strain gull/MR04_RV/HUN/2014 belongs to the NSP3_RV superfamily and has a similar structure to protein cd20714 (E-value: 6.20e-23). A total of 30 aa residues of the RNA-binding site were detected and within and after this site, three distinguished aa motifs (PKR, NLEKEVRMLR and NSTSDLKLS) could be identified compared to the reference protein.

Each nucleotide sequences of strain gull/MR04_RV/HUN/2014 segments were sent to the NCBI BlastX to search the translated nucleotide query in the clustered protein database (S2 Table). All segments have high (more than 85%) nucleotide identity to ruddy turnstone RV isolate MW05 (MH453863-MH453873) in RVG with significant query cover, but four segments have low sequence identity to other types of RVs (S2 Table). The **segment 4** encoding the VP4 (VP5/VP8) protein(s) has **40.54%** aa identity (query cover: 97%) to RVB species strain RVB/Goat-wt/USA/Minnesota-1/2016 (KY689690/ASV45170) as the best match, followed by the closest RVG strain chicken/03V0567/DEU/2003 (JQ920006/ AFL91895) with 39.45% identity (query cover: 96%). The genetic analysis of **segments 5** (NSP1), **9** (VP7) and **10** (NSP4), although the segment identities were the highest with homologue segments of RVG strains RVG/turkey-wt/USA/Minnesota-1/2016 (KY689680/ASV45160), Shackleton virus (MT025062/QIS87933) and DRVG/G018-19/Australia/2019 (MW388702/UAJ21477), respectively, with a very low nt and aa identities (S2 Table).

**Fig 1:**
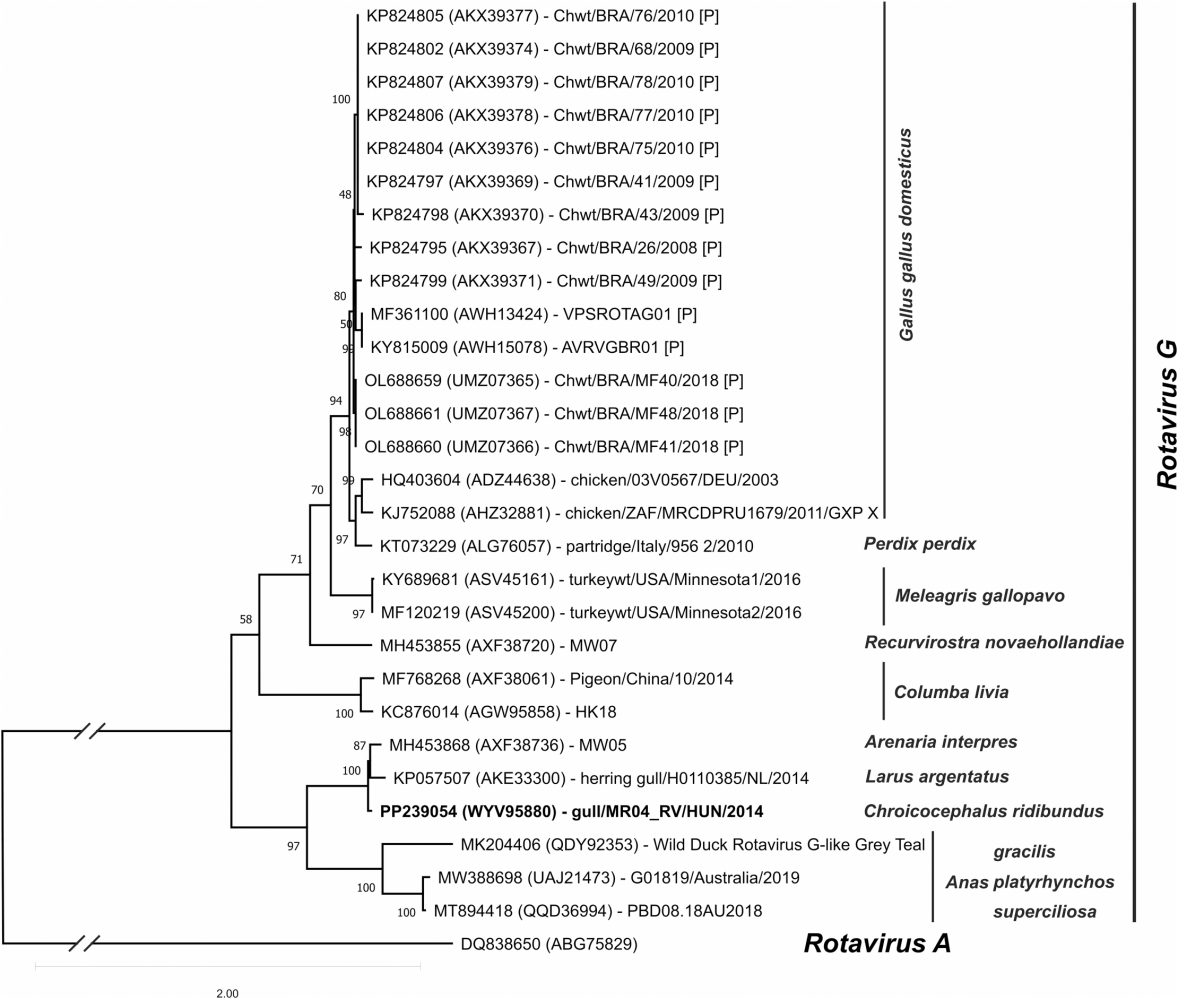
Phylogenetic comparison of segment 6 encoding VP6 protein. Phylogenetic analysis of the internal capsid protein VP6 encoded segment 6 of strain gull/MR04_RV/HUN/2014 (PP239054, PP239059) rotavirus (RV) and all complete and partial amino acid coding nucleotide sequences of group G rotaviruses available in the NCBI GenBank database were used for the VP6 analysis. The nucleotide sequences were aligned using the Multiple Alignment using Fast Fourier Transform (MAFFT) v7.525 (2024/Mar/13) [33] and then analysed using IQ-TREE software with the following parameters: -m MFP, -alrt 1000, -T AUTO, -bb 100 [34,35,36]. According to the Bayesian Information Criterion (BIC) score the best-fit model for the VP6 dataset was TIM2 + F + R3 and for the NSP5 dataset was TIM2 + F + I + G4 models, respectively. Both the SH-aLRT test and the ultrafast bootstrap (UFBoot) values were set up to 1000 in each analysis, then the maximum likelihood consensus tree was used. The nucleotide, the amino acid accession identification numbers and the strain/isolate name of the sequences were indicated on the phylogenetic tree. The study strain gull/MR04_RV/HUN/2014 (PP239054, PP239059) sequence is marked in bold. [P]: partial sequence.

The nt sequence analyses of the VP6 inner capsid protein of the study strain showed that it is genetically most closely related to members of RVG species, and the closest relative is a partial 570nt/190aa long VP6 sequence of the strain herring gull/H0110385/NL/2014 (KP057507, AKE33300) isolated from an FFPE cloacal bursa tissue of host *Larus argentatus* in the Netherlands in 2014 (Fig 1). The second closest related sequence is the Ruddy turnstone rotavirus isolate MW05 (MH453863- MH453873) isolated from a combined oropharyngeal and cloacal swab in the King Island, Australia in 2014 (Fig 1). The phylogenetic analysis of the further segments of the study strain, each segment showed a relative relationship with the corresponding segments of the isolate MW05, however, segments 4, 5, 9 and 10 are more homologous to members of the RVB group. The list of RV sequences included in this study is collected in S3 Table and the detailed phylogenetic analysis of each segment can be found in S3 Fig.

**Fig 2:**
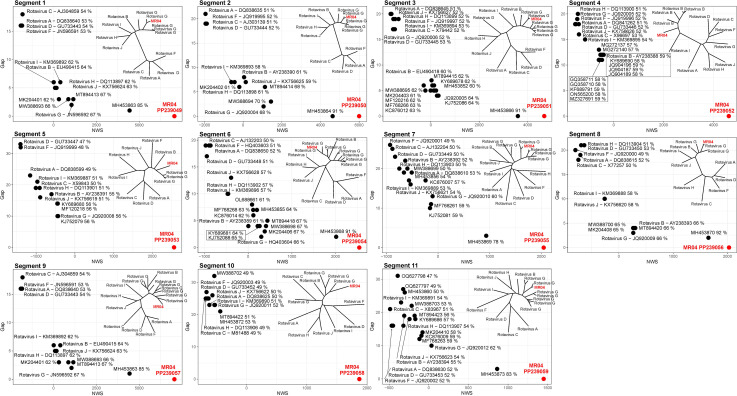
Phylogenetic and Needleman-Wunsch analysis of the study strain gull/MR04_RV/HUN/2014 (PP239049-PP239059) rotavirus (RV) segments (1–11) and corresponding segments of reference, closest relative RV strains. The main diagrams (segments 1–11) show the relationship between the reference or closest related sequences aligned to the segments of the study strain sequence using the Needleman-Wunsch (NW) Global Alignment method based on the NW scores (X-axis: NWS), the alignment GAP (Y-axis: GAP) and the percent of identity (points). The phylogenetic position of the study strain has also been indicated in the small figure. A detailed phylogenetic analysis of each segment can be found in S3 Fig.

By aligning all the genome segments of the strain gull/MR04_RV/HUN/2014 to the corresponding segments of reference and the closest related RV strains using the Needleman-Wunsch alignment method, low similarity values were obtained, especially for those of the diverse four segments 4, 5, 9 and 10 mentioned above (Fig 2) [43].

**Fig 3:**
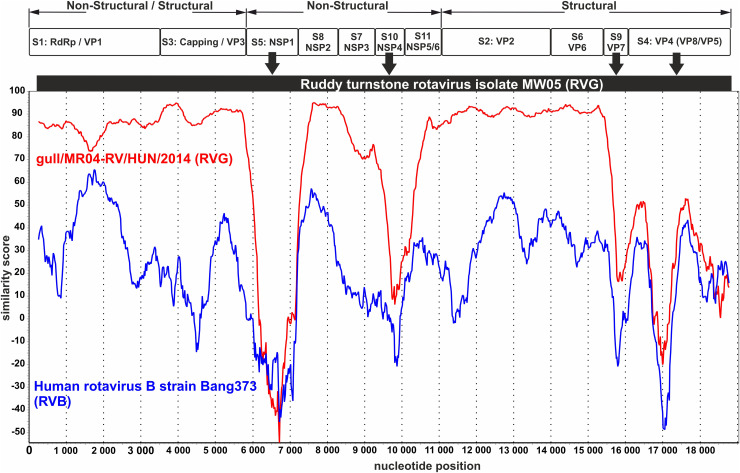
The similarity analysis of the study strain gull/MR04-RV/HUN/2014 (RVG), the closest relative ruddy turnstone rotavirus isolate MW05 (RVG) and human rotavirus B strain Bang373 (RVB) with SimPlot software [ 37]. For the analysis, the entire genomes of the study strain gull/MR04-RV/HUN/2014 genome segments (PP239049-PP239059) rotavirus, the closest relative ruddy turnstone rotavirus isolate MW05 (MH453863-MH453873) and human rotavirus B strain Bang373 (EU490415, EU490418, AY238384, AY238385, AY238388- AY238394) were concatenated and aligned using Multiple Alignment using Fast Fourier Transform (MAFFT) method (https://www.ebi.ac.uk/jdispatcher/msa/mafft). The aligned nucleotide sequences were analysed with the SimPlot program based on the following setup: Window: 500 bp, Step: 20 bp, GapStrip: On, F84 (“Maximum Likelihood”), T/t: 2,0). The segments coding for non-structural and capsid proteins (with frames) and possible reassortant segments (with arrows) are marked in the figure. RVG: rotavirus group G, RVB: rotavirus group B, S1–S11: segment 1–11.

Analysis of the merged segments of the strain gull/MR04_RV/HUN/2014, the ruddy turnstone RV isolate MW05 (RVG) and the human rotavirus B (RVB) strain Bang373 yielded surprising results. In the analysis, segments similar to the RVG strain homologated to the RVG reference sequence, while segments with low identity values (5: NSP1 and 10: NSP4, 9: VP7 and 4: VP4) showed surprising similarity to the corresponding segments of human rotavirus strain Bang373 (RVB), which may be an exciting example of reassortment between RV species (RVG/RVB) (Fig 3).

### Characterization of Avastrovirus 2 (AAstV-2) strain gull/MR04_AAstV/HUN/2014

**Fig 4:**
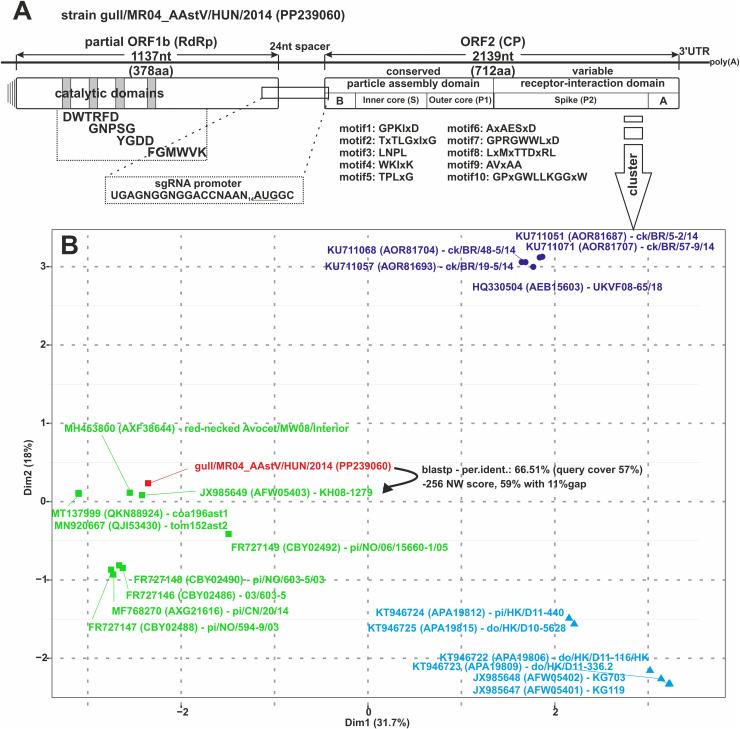
Genome organization (A) and Cluster analysis (B) of the Avastrovirus 2 (AAstV-2) strain gull/MR04_AAstV/HUN/2014 (PP239060) astrovirus. A) The genome organization of the partial sequence of gull/MR04_AAstV/HUN/2014 astrovirus was constructed based on the Duck astrovirus strain CPH (KJ020899). The conserved nucleotide sgRNA signal and the conserved amino acid motifs in the ORF1b and ORF2 proteins are highlighted. B: basic amino acids, A: acidic amino acids. B) The cluster analysis of the study strain gull/MR04_AAstV/HUN/2014 (PP239060) and the representative strains in AAstV-2 group 6. The cluster analysis was done with an R script (in this study) and “seqinr”, “msa”, “cluster”, “factoextra”, “randomcoloR”, “ggrepel” and “ape” packages using R/RStudio. The script of the analysis can be provided as requested. Arrow shows the most closely related virus sequence AAstV-2 isolate KH08-1279 (JX985649) with the BlastP identity and the Needleman-Wunsch (NW) Global alignment score results. The study strain is coloured red. The detailed phylogenetic analysis of avian astroviruses can be found in the appendix S4 Fig.

The determined partial genome of astrovirus strain gull/MR04_AAstV/HUN/2014 (PP239060) is 3,448nt long (partial ORF1b is 1,137nt/378aa and the full-length ORF2 is 2,139 nt/712aa) (Fig 4A). The partial ORF1b encoded polyprotein contains the putative aa motifs (DWTRFD, GNPSG, YGDD, and FGMWVK) of the catalytic domain of the astroviral RdRp enzyme [44,45]. The sub-genomic RNA nt sequence motif (UUUGGAGNGGNGGACCNAAN_4-11_AUGNC) is identifiable in the study strain, the modified sequence of which is as follows UGAGNGGNGGACCNAAN_14_AUGGC (N: any of four nucleotides, the ORF2 initiation codon is underlined). The ORF1b is non-overlapped with ORF2, a 24nt-long spacer is observed, but the “CCGA” AAstV pentamer [46] is not present. The conserved nt sequence of stem-loop II (s2m) mobile genetic elements could not be identified in either the partial ORF1b or the 3’UTR. The closest related sequence of the partial ORF1a in the GenBank database was an *Astroviridae* sp. isolate prf038ast1 (MT138011) sequence from a bird metagenome with 66.55% nt identity (query cover: 77%) and the ORF1b protein of ruddy turnstone astrovirus isolate Prototype-RtAstV (MK189093, QCP68856) sequence with 64.55% (query cover: 100%) aa identity.

The ORF2 capsid protein of the strain gull/MR04_AAstV/HUN/2014 is likely to be an astrovirus capsid protein based on the NCBI CDS and BlastP identity analysis. The ORF2 protein is separated into a conserved particle assembly domain (AD) and a variable receptor-interaction domain (RID) (Fig 4A). Based on the NCBI CDS and a comparative sequence analysis with the reference/representative types of avian nephritis viruses (ANV) [46], the first 390 aa elements build the inner and outer ORF2 capsid core contain conservative elements/motifs while the spike of the ORF2 protein is variable and has no detected conserved motif and has low sequence identity. Examining the nt sequence encoding the ORF2 capsid protein, the closest related sequence was an *Astroviridae* sp. isolate tom152ast2 (MN920667) sequence from a bird metagenome with 66.51% nt identity (query cover: 52%) and the corresponding protein of Avastrovirus 2 isolate KH08-1279 (JX985649, AFW05403) from an Ardeola bird sp. with 52.76% aa identity (query cover: 83%) (Fig 4B). Based on the phylogenetic analysis performed on all capsid (complete and partial) proteins (N = 821) of genus *Avastrovirus* (taxid:249589) the study strain belongs to the avian nephritis virus (ANV) group in Avastrovirus 2 (AAstV-2) (S4 Fig). According to the genetic identity and the aa-based cluster analysis, the study strain is closely related to isolate KH08-1279 (JX985649, AFW05403) and strain red-necked avocet/MW08/Interior (MH453800, AXF38644) viruses which are classified in group 6 of ANVs (Fig 4B, S4 Fig) [46].

Using the screening primer pairs (MR04-GVP6-SCR-R/ MR04-GVP6-SCR-F; MR04-AvAst2-SCR-R/ MR04-AvAst2-F) neither RV nor astrovirus could be found in the further faecal samples of common black-headed (N=4) and yellow-legged (N = 2) gulls.

## Discussion

The species-rich *Laridae* bird family includes the genera gulls, terns, noddies, skimmers and kittiwakes, which are primarily seabirds which are widespread in the world, but species that can also be found in Western Europe, e.g.,: European herring gull (*Larus argentatus*) and in Northern Europe, e.g.,: common gull (*Larus canus*) and in Central Europe, e.g.,: black-headed (*Chroicocephalus ridibundus*) and yellow-legged (*Larus michahellis*) gulls including Hungary [1]. Considering their lifestyle, these birds may come into close contact with humans, and the viral metagenomic analysis of their excrement can provide useful information about the prevalence of viruses excreted in faeces. Viral metagenomics using NGS methods can reveal many potential new pathogens, however, this discovery is only a meaningless and unmanageable data set in the absence of further detailed analysis [28,43].

In this study, after evaluating the mass sequencing data, we focused on viral sequences occurring in vertebrates, among which viral families *Sedoreoviridae* and *Astroviridae* were interesting for deeper investigation. Due to the low percentage of reads (less than 0.3% per virus family) and the uncertain taxonomic classification (less than 30% identity) of reads belonging to the families *Coronaviridae*, *Picobirnaviridae*, *Retroiridae*, *Caliciviridae* and *Picornaviridae*, were not analyzed in this study. The data obtained during mass sequencing was filtered using the Kaiju method based on the NCBI nr protein database (accessed 03.27.2023.) and then confirmed using the diamond method. The obtained reads were aligned to the closest related sequence, and then verified with sequence-specific primers, after we reported on a potential interspecies reassortant RVG strain gull/MR04_RV/HUN/2014 and an avian astrovirus (AAstV-II) strain gull/MR04_AAstV/HUN/2014.

The RV identified in the black-headed gull faecal sample is an exciting finding from several points of view. Firstly, according to the rule defined by the International Committee on Taxonomy of Viruses (ICTV) *Sedoreoviridae* study group, viruses whose VP6, encoding in segment 6, amino acid sequence has more than 53% identity belong to the same species within the RV genus (https://ictv.global/report/chapter/sedoreoviridae/sedoreoviridae/rotavirus) [3]. Based on this criterion, the black-headed gull RV strain gull/MR04_RV/HUN/2014 belongs to the genus *Rotavirus* and species *Rotavirus G* (RVG), but with a low (66%) nucleotide identity value. Secondly, however, most of the RV conserved motifs identified by Trojnar et al. [8] in the functionally important proteins (RdRp/VP1, Cap/VP3, NSP2) were present in the study RV strain, but most parts of the viral genome show significant differences from members of the RVG, especially the segments encoding the NSP1, NSP4, VP7 and VP4 proteins.

The first RVG species was identified from chickens (*Gallus gallus*) [47] as potentially causing runting and stunting syndrome [48] and then an RVG species was also described from turkeys (*Meleagris gallopavo*) [49]. To date, RVG species have been identified in many other species of wild birds, such as ducks (*Anatidae*), pigeons (*Columbidae*), partridges (*Perdicinae*), gulls (*Laridae*), and interestingly pigs (Sus scrofa domesticus) (Fig 1). Based on the analysis of segment 6 encoding the VP6 capsid protein, the closest relative was the RV identified from herring gull in the Netherlands in 2014. Unfortunately, only the partial VP6 sequence of isolate herring gull/H01-10385/NL/2014 is available in the GenBank database [50], so we could not use this isolate for a deeper analysis covering additional segments. Comparing the corresponding regions of viruses with a complete genome, the closest relative of the study strain was the RVG isolate MW05 (MH453868) described from ruddy turnstone waterfowl in Australia in 2014. Until August 2023, RVG types had been described only from avian species, but Dias and colleagues found RVG group rotavirus strains RVG/Pig-wt/BRA/BP52/2016 (OR067849) and RVG/Pig-wt/BRA/BP56/2016 (OR067850) in pigs in Brazil in 2016 (unpublished, only sequences are available). The RVG viruses identified in pigs are closely related to RVG types identified in chickens, including the reference sequence of the RVG group strain chicken/03V0567/DEU/2003 (NC_021587). Unfortunately, only the partial NSP5 portion of the two pig RVG strains mentioned above is available in the GenBank database, which we could not use for our VP6-based sequence analysis.

In a detailed analysis, the segments encoding additional structural and non-structural proteins, very low nt identities (with a high overlap value) were observed, in segments 4, 5, 9 and 10 compared to any of the known corresponding RV sequences. Among these four segments, segments 5 and 10 encode non-structural NSP1 and NSP4 proteins respectively, of which the nearly 500-aa-long NSP1 protein has a potential role in inhibiting the host’s innate immunity by breaking down the key factors required to activate host interferon production [51]. The aa similarity of the NSP1 protein to members of RVG proved to be at most 35%, however, the phylogenetic analysis of this segment with members of other RV groups showed a clear relationship with the human RVB strain Bang373 (RVB) (Fig 2). The NSP4 of segment 10 is a relatively small, 175-aa-long protein that functions as a viroporin [52]. The nt sequence of segment 10 had no significant match with any of the known RVG viruses and the aa level has the closest to RVG strain DRVG/G018-19/Australia/2019 (UAJ21477) but with an extremely low aa identity value. Interestingly, in the nt level the NSP4 protein of the study strain shows significant identity (with no significant query cover) with RVB strain RVB/Pig-wt/VNM/14176_8/NSP4 (KX362398) described in porcine [53] (S2 Table). The VP7 protein of segment 9 shows low nt and aa identity to RVG viruses, while VP4 encoded in segment 4 has no relationship to RVG viruses at either nt or aa level. In contrast, the VP4 is related to the corresponding protein of the RVB strain JN311 (KU562898) isolated from human stool in Bangladesh [54]. The low-level nt and aa identities observed in segments 4, 5, 9 and 10 of the study strain compared to RVG lead to the conclusion that these segments have originated from a currently unknown RV related distantly to RVB which could make the study RV strain a potential reassortant virus and may be able to infect and replicate successfully among in RVB viruses. Based on the analysis of the concatenated nucleotides of all segments of the study strain gull/MR04_RV/HUN/2014, and the closest relative partners ruddy turnstone RV isolate MW05 (RVG) and human rotavirus B strain Bang373 (RVB), it can be assumed that the RV identified in this study is, in fact, an interspecies (RVG/RVB) reassortant virus capable of infecting birds (RVG), rodents (RVB), pigs (RVB) and possibly the human (RVB) population.

Group B rotaviruses (RVBs) have been identified in various species, including humans [54; 55], swine [56–58], bovines [58], caprine [59], equines [58,60,61], lambs [62], and goats [62], and rodents [63]. Human RVB infections or outbreaks have been reported in Asia, specifically in Nepal [64], India [65,66], and Bangladesh [54,67], as well as in South Africa [68]. In other parts of the world, swine are considered the primary reservoir for RVBs. But in Europe, no human RVB infections or outbreaks have been documented. However, a few studies have identified RVBs in animal hosts, particularly swine [56–58], bovines [58], equines [58], lambs [62], and goats [62], albeit in limited geographic regions. Despite these findings, there is no formal epidemiological surveillance system in the EU/EEA for monitoring rotavirus infections or circulating strains, as noted by the European Centre for Disease Prevention and Control (ECDC) (https://www.ecdc.europa.eu/en/rotavirus-infection/facts). Most available data on RVBs in Europe come from isolated studies, highlighting the need for further research to better understand the significance of RVB in animal and human diarrheal diseases.

Point mutations, intragenic [13] and intergenic (inter-lineage/-sub-lineage) recombination [14,15,16] and reassortation (between single or multiple genome segments) [17] evolutionary mechanisms as a whole are the driving forces of strain variation that can facilitate the adaptation of the segmented RV with a double-stranded RNA genome to the host [11–13,69]. However, point mutations and intragenic and intergenic recombination are well-studied mechanisms, as a new observation, a recombination phenomenon was also described between segments of different RV species in two cases [18,19]. Both studies reported partial or complete replacement of the NSP3 protein (segment 7) of porcine RV belonging to the RVH group with the corresponding segment of porcine RV belonging to the RVC species, which was the first evidence of interspecies recombination. In 2017, two turkey RVs (strains Turkey-wt/USA/Minnesota-1/2016 and Turkey-wt/USA/Minnesota-2/2016) were described in the RVG group, a possible recombination event was detected in VP6 and NSP3 proteins compared to the member of the RVB human strains RVB/Human-wt/BAN/Bang117/2002 (GU391305) and RVB/Human-wt/JAP/MMR-B1/2007 (GU370059) corresponding partial regions [70].

The mechanism of reassortment has been observed between the same RV species, especially in group A RVs, is also a well-known phenomenon [71,72], where the two most important neutralization antigens VP4 (encoded on segment 4) and VP7 (encoded on segment 9) proteins, as well as NSP4 (encoded on segment 10) and NSP5/6 (encoded on segment 11) proteins, are most likely to be replaced [73]. The VP4 binds the virion to the host’s epithelial cell receptors and delivers it into the host cell [74]. Presumably, the binding of RVs and their entry into the host cell must be the result of multiple consecutive contacts between the outer capsid proteins VP4 and VP7 and cell receptors, in which a significant change can modify the virulence of the virus, and even widen the possible host spectrum or other host specificity can result [74]. The NSP4 protein is important for viral morphogenesis and has enterotoxin activity [73], and the NSP5/6 proteins are useful for tracing the species origin of RVs [73].

The molecular process of reassortment is not fully understood, but, likely, reassortment does not require physical proximity to the parental genomes and there is growing evidence to support the selective packaging model, that uses a signal-driven selective segment incorporation process to shape newly generated viral particles, as seen in the influenza virus [69,75–77]. The virus assortment is likely coordinated by RNA–RNA interactions and a selective packaging signal/ cis-acting element according to the strategy of the “Concerted model” [78]. The conserved sequences at the 5’ and 3’ ends of the rotavirus segments are so-called “cis-acting elements (CRE)” with different functional content essential for selective packaging [78], but the CRE at the 3’end serves as a critical polymerase recognition element and provides a template for genome replication [78].

In our study, the genetically and phylogenetically analysed strain gull/MR04_RV/HUN/2014 is closely related to RVG group viruses based on the proteins VP1-VP4, VP6, NSP2, NSP3, NSP5, while based on the non-structural proteins NSP1, NSP4 and the structural proteins VP7, VP4, it is more related to the human RVB strain Bang373. The RVG species described in this communication is presumably an interspecies reassortant virus, which has been supported by several indirect observations so far: **a**) the RVG and RVB virus species belong to the same RVB-like clade (clade 2) and are juxtaposed in the phylogenetic analysis of all 11 segments as seen in S3 Fig [6,9] and phylogenetically closely related viruses are genetically similar and, in case of a co-infection, are more likely to produce infectious virus particles by reassortation. **b**) Comparing the 5’ and 3’ consensus ends of the study strain, it is worth directing attention to segments 2 (VP2), 4 (VP4), 5 (NSP1) and 10 (NSP4) where a high degree match with the consensus elements of the RVB group was found (S2 Fig). **c**) Two cases of putative recombination events between turkey RVG and human RVB species have been reported, involving the segments encoding VP6 and NSP3 proteins [70], in the study strain there was not a pointwise or intermittent excision and replacement of certain parts of the segments, but a replacement of entire segments could have happened. **d**) Several cases of rotaviruses belonging to different RV groups have been described that were present in the same host, causing co-infection [79–81]. Interestingly, in a Chinese study, a survey of pig herds showed the co-occurrence of RV belonging to other species in many cases, where some type of co-infection was found in 40% of the examined samples [81]. Among the test results, the most surprising result was the 2% quadruple (RVA+RVB+RVC+RVH) RV infection [81]. **e**) Finally, the RVG type has been detected only in avian species but it has been described in 2 cases in pigs (unpublished, only nucleotide sequences are available).

The co-circulation of different RV species in the same host species can facilitate the exchange of genes or even segments between different RV strains. Considering the lifestyle of the black-headed gulls described in this study, it is likely that they could have been infected with the RVB virus in human (contaminated with faeces) waste (food?) or in an intermediate host (swine?), which could have recombined during pathogenesis with the RVG virus typically found in birds.

The astrovirus identified in the analysed black-headed gull faecal sample phylogenetically belongs to the genus *Avastrovirus* species Avastrovirus 2 (AAstV-2) and avian nephritis viruses (ANVs). Unfortunately, due to the small amount of faecal sample collected from black-headed gull and the importance of the RV presented in this study, it was not possible to determine the complete genome of the strain gull/MR04_AAstV/HUN/2014. Unfortunately, only the partial ORF1b and the complete ORF2 capsid sequences, which exhibited properties characteristic of the AAstV (the nt sequence of the conserved sgRNA promoter between ORF1b/ORF2, the key conserved aa motifs characteristic of the polymerase enzyme) was analyzed. The classification of AAstVs is currently (since 2009) based on the hosts from which they have been isolated and the re-grouping of the known sequences is currently underway, with analysis of the entire capsid protein of the virus likely to be one of the pillars (https://ictv.global/report_9th/RNApos/Astroviridae). Currently, there are three official AAstV species (AAstV 1-3) and within these, many viral sequences await classification [4]. Based on the survey by Kariithi et al. [46], the AAstV-2 species, avian nephritis viruses (ANVs) group is subdivided into 11 additional sub-genotypes (groups 1–11). The nt genetic distance within each virus sub-genotype group varies between 0.204 and 0.284. The mean value calculated in group 6 is 0.418 [24,25,46], which raises the possibility of forming further groups, and we calculate that four more groups (group 6/A: 0.36, group 6/B: 0.43, group 6/C: 0.19, and group 6/D: 0.22) could be formed and visualized in S4 Fig, among which the gull/MR04_AAstV/HUN/2014 study strain presented here (group 6/B) would also occupy an important place.

The astrovirus included in the study is phylogenetically related to strain KH08-1279 virus isolated from pond herons (*Ardeola sp.*) in China and strain isolate AstV/Red-necked Avocet/MW08/Interior virus isolated from red-necked avocet (*Recurvirostra novaehollandiae*) in Australia. Both astroviruses were described in the faecal samples of waterfowl whose related species, e.g.,: little egret (*Egretta garzetta*), and pied avocet (*Recurvirostra avosetta*) also have known populations in Hungary, and this observation assumes that Hungarian herons, gulipans, and mostly gulls can be potential carriers and spreaders of AAstVs due to their migratory and aquatic lifestyle.

In conclusion, the wild bird populations are greatly affected by urbanization, which narrows the original habitats of waterfowl, forcing the majority of species to adapt to urban lifestyles. Paradoxically, gulls have been able to adapt to urban life, where landfills and polluted waterways allow them an expanded food source and at the same time transmission of human pathogens. Due to the very small number of study samples, likely, one piece of the whole puzzle regarding the diversity of RVs and astroviruses is found in nature. Further molecular surveillance for gulls or other related species adapted to human environments (crows, pigeons, etc.) and even more waterfowl can provide material for the discovery of already known or novel RVs, astroviruses and other novel viruses with RNA genomes.

## Supporting information

S1 Fig Classification of reads derived from viral metagenomic analysis. (DOCX)

S2 Fig Comparison of 5’- and 3’-terminal non-coding regions (5’/3’ NCRs) sequences. (DOCX)

S3 Fig Phylogenetic analysis of the corresponding segments of the study strain, closest related representative sequences and reference rotavirus strains. (DOCX)

S4 Fig Phylogenetic and Cluster analysis of avastroviruses.
(DOCX)

S1 Table The genome organization of group G rotavirus strain gull/MR04-RV/HUN/2014.
(DOCX)

S2 Table Nucleotide and amino acid identity comparison of strain gull/MR04-RV/HUN/2014.
(DOCX)

S3 Table List of rotavirus sequences included in the analysis.
(DOCX)

S1 File Web References. (DOCX)
