# Supplementary material for: Identification of a potential interspecies reassortant rotavirus G and avastrovirus 2 co-infection from black-headed gull (Chroicocephalus ridibundus) in Hungary
Source: PLoS One. 2025 Mar 24;20(3):e0317400. doi: 10.1371/journal.pone.0317400 (PMC11932466; doi:10.1371/journal.pone.0317400)
Supplement: S1 Fig — (DOCX) [file pone.0317400.s001.docx]

**S1 Fig. Classification of reads derived from viral metagenomic analysis.**

Grouping of reads obtained metagenomic analysis following bioinformatic interpretation of data. In the case of viral families, the most probable host was determined based on the NCBI taxonomic database ("host", "viral host") [1]. The reads classified into virus families were grouped according to host organisms and displayed on a bar chart, where the individual hosts are displayed on the x-axis while the log_10_ of the number of reads is displayed on the y-axis. The number of reads belonging to each family is indicated on the diagram. The diagram was created using the ggplot2 library in the R/RStudio program.


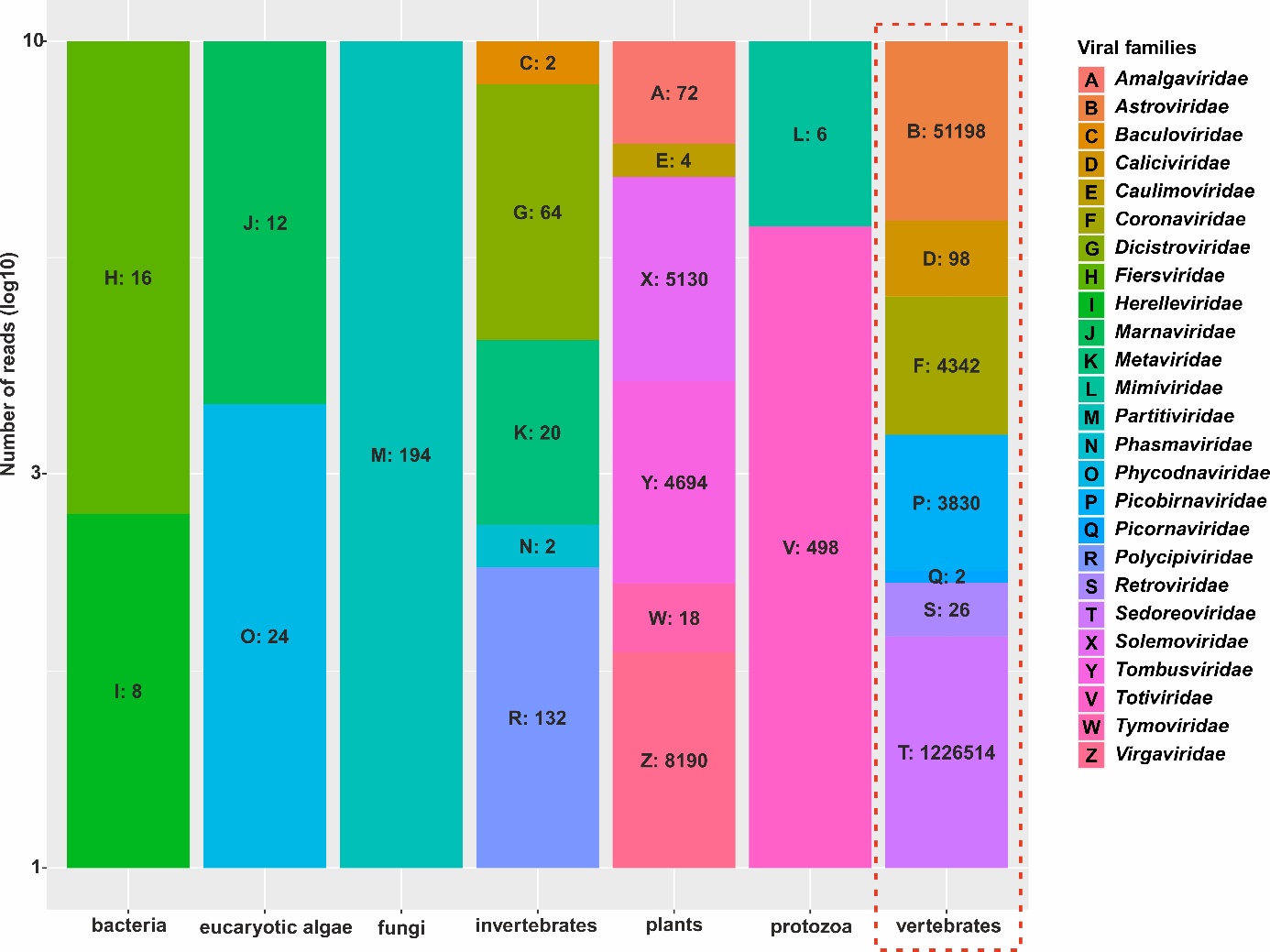


References

1. Schoch CL, Ciufo S, Domrachev M, Hotton CL, Kannan S, Khovanskaya R, et al. NCBI Taxonomy: a comprehensive update on curation, resources and tools. Database (Oxford). 2020; baaa062. doi: 10.1093/database/baaa062
