# Supplementary material for: Identification of a potential interspecies reassortant rotavirus G and avastrovirus 2 co-infection from black-headed gull (Chroicocephalus ridibundus) in Hungary
Source: PLoS One. 2025 Mar 24;20(3):e0317400. doi: 10.1371/journal.pone.0317400 (PMC11932466; doi:10.1371/journal.pone.0317400)

**S2 Fig.** **Comparison of 5′- and 3′-terminal non coding region (5'/3' NCR) sequences.**

Comparison of 5′- and 3′-terminal non coding region (5'/3' NCR) sequences of group G rotavirus strain gull/MR04-RV/HUN/2014 genome segments (PP239049- PP239059, MR04) to those of RVA-like clade (clade 1): group A (RVA), C (RVC), D (RVD) and F (RVF) and RVB-like clade (clade 2): group G (RVG), B (RVB), I (RVI), J (RVJ) and H (RVH) reference rotavirus sequences. Compared to the own sequence, the matching nucleotides are marked with a dot, and the closest identical sequence(s) is/are marked with a dashed blue frame while the changes are marked with the corresponding nucleotide. RVA: RVA/Simian-tc/ZAF/SA11-H96/1958/G3P5B[2]; RVC: RVC/Human-tc/GBR/Bristol/1988/G4P[2]; RVD: RVD/Chicken-wt/DEU/05V0049/2005/GXP[X]; RVF: RVF/Chicken-wt/DEU/03V0568/2003/GXP[X]; RVG: RVG/Chicken-wt/DEU/03V0567/2003/GXP[X]; RVB: RVB/Human-wt/BGD/Bang373/2000/GXP[X]; RVI: KE135/2012; RVJ: BO4351/Ms/2014; RVH: RVH/Human-tc/CHN/NADRV-J19/1997/GXP[X].


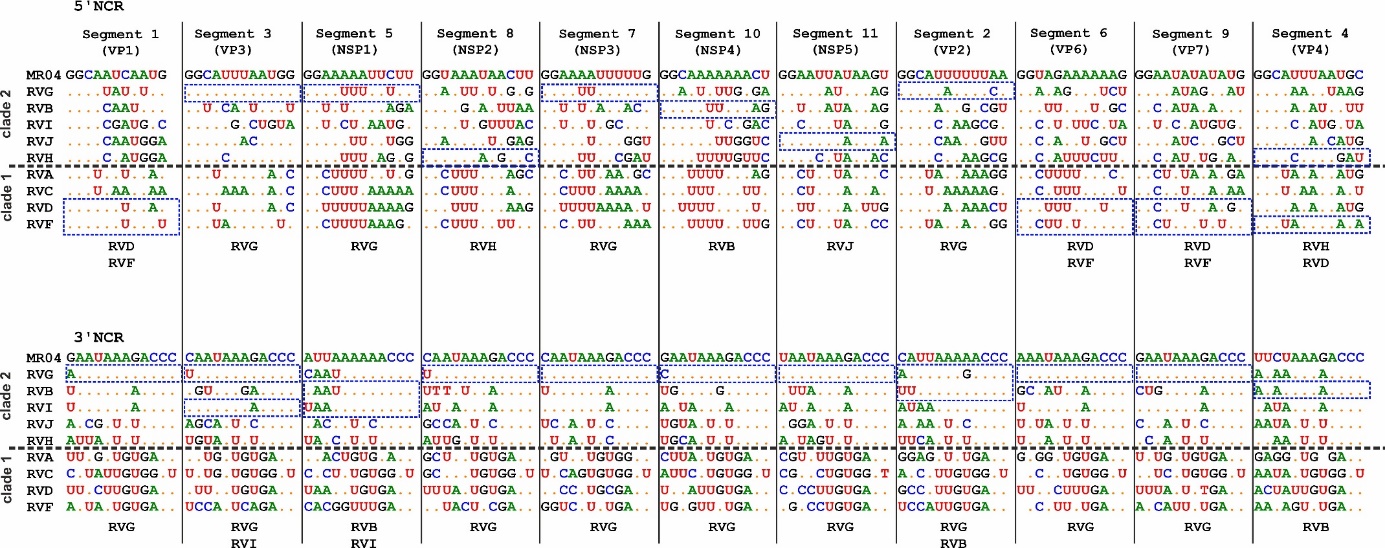

Supplement: S2 Fig — (DOCX) [file pone.0317400.s002.docx]
