# Supplementary material for: Identification of a potential interspecies reassortant rotavirus G and avastrovirus 2 co-infection from black-headed gull (Chroicocephalus ridibundus) in Hungary
Source: PLoS One. 2025 Mar 24;20(3):e0317400. doi: 10.1371/journal.pone.0317400 (PMC11932466; doi:10.1371/journal.pone.0317400)
Supplement: S3 Fig — (DOCX) [file pone.0317400.s003.docx]

**S3 Fig. Phylogenetic analysis of the corresponding segments of the study strain, closest related representative sequences and reference rotavirus strains.**

The deduced amino acid sequences were aligned using the MEGA11/Muscle method with default settings and the aligned amino acid sequences were tested with MEGA11/Find Best DNA/protein model search [1]. The statistical method determined by the lowest BIC scores were chosen as the basis for the phylogenetic analysis. Phylogenetic analysis was performed using the Maximum Likelihood (ML) statistical method using LG with Freqs (+F) model with Gamma distributed rates in segment 1, in segment 2, in segment 4, in segment 6-10, WAG with Freqs (+F) model with Gamma distributed rates in segment 3, in segment 5, and JTT with Freqs (+F) model with Gamma distributed rates and Invariant sites segment 11. In the phylogenetic trees, the group G rotavirus strain gull/MR04-RV/HUN/2014 genome segments (PP239049- PP239059) was marked with a box.

segment 1 / VP1 (Pol)


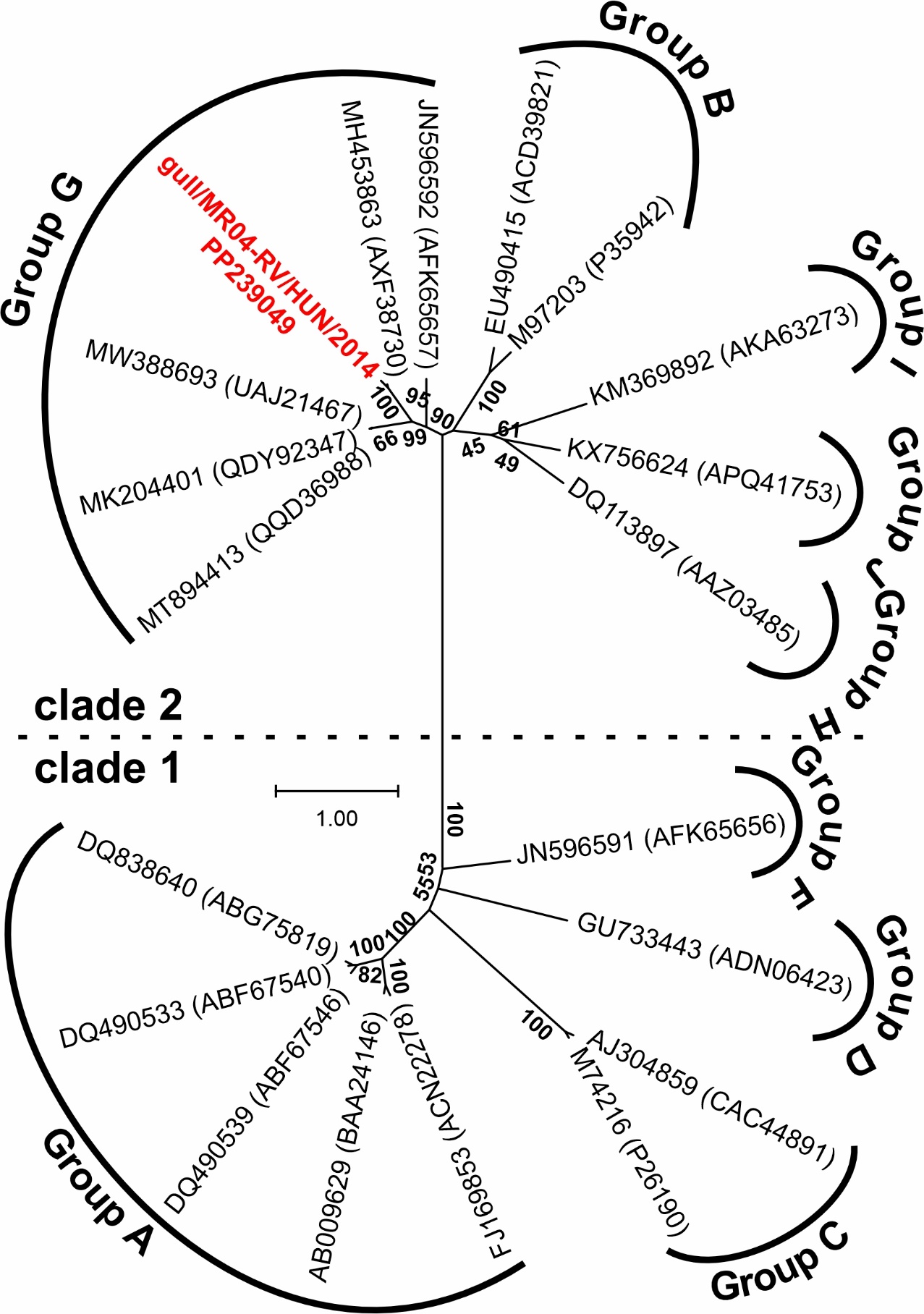


segment 3 / VP3


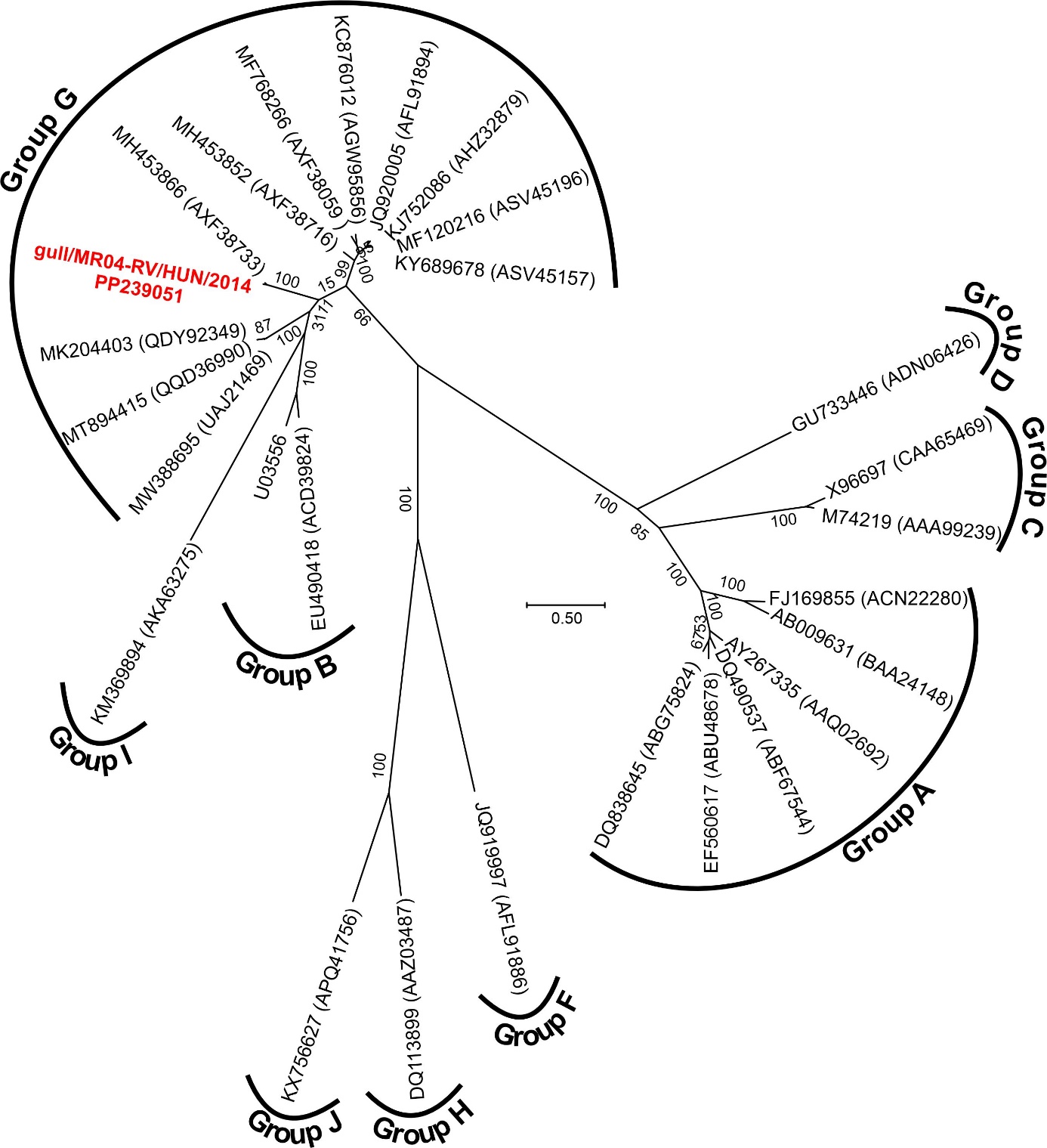


segment 5 / NSP1


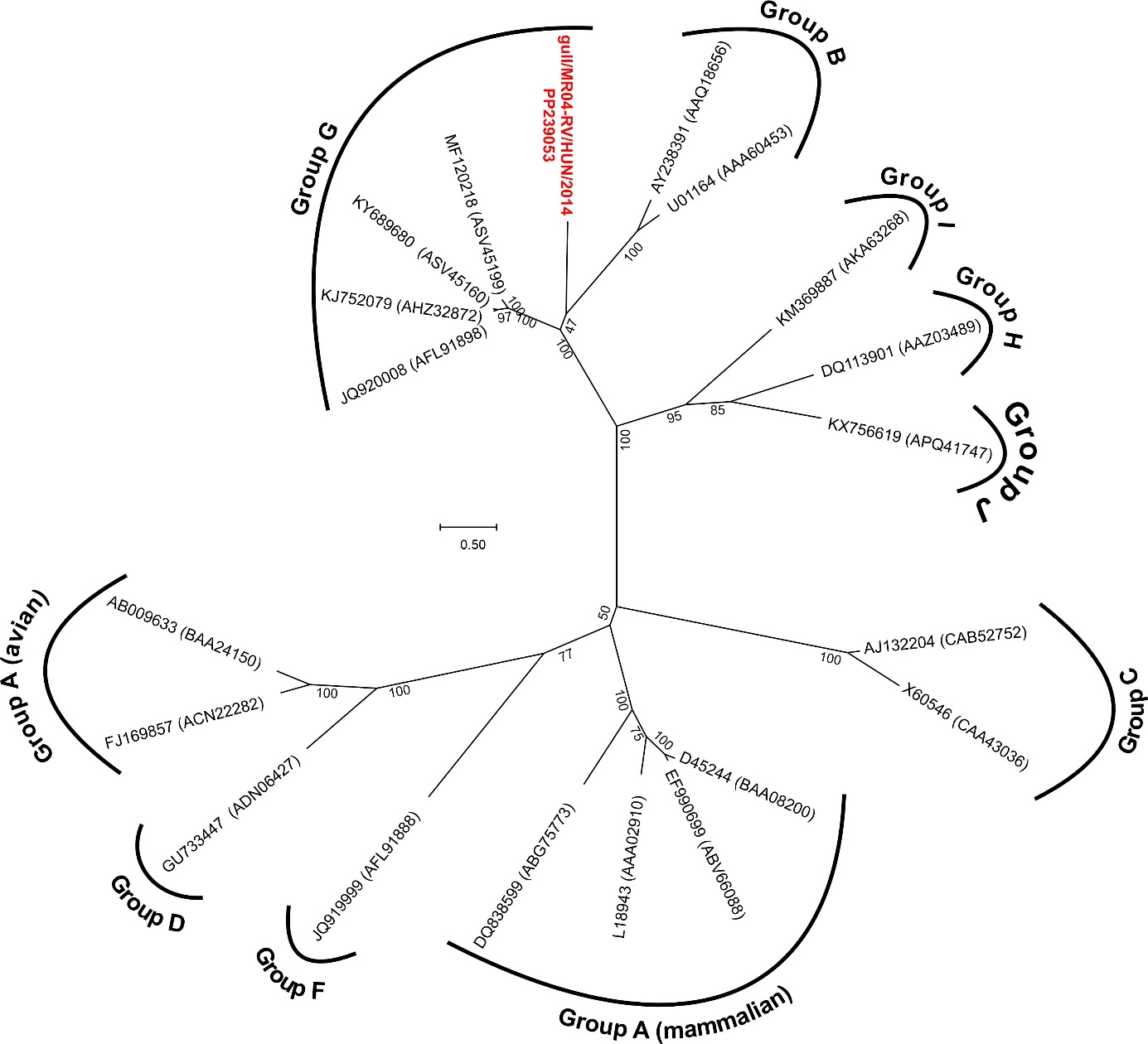


segment 8 / NSP2


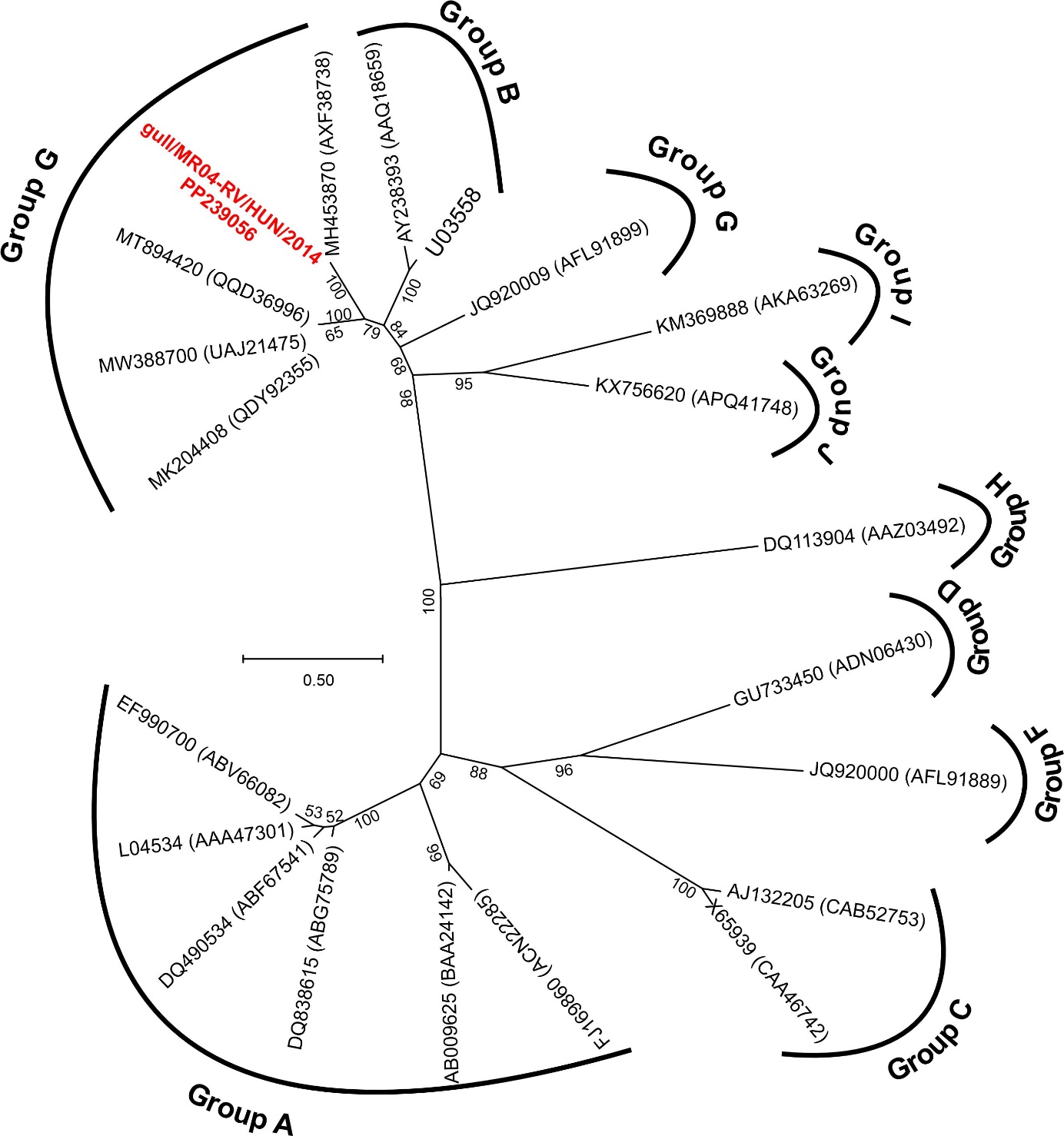


segment 7 / NSP3


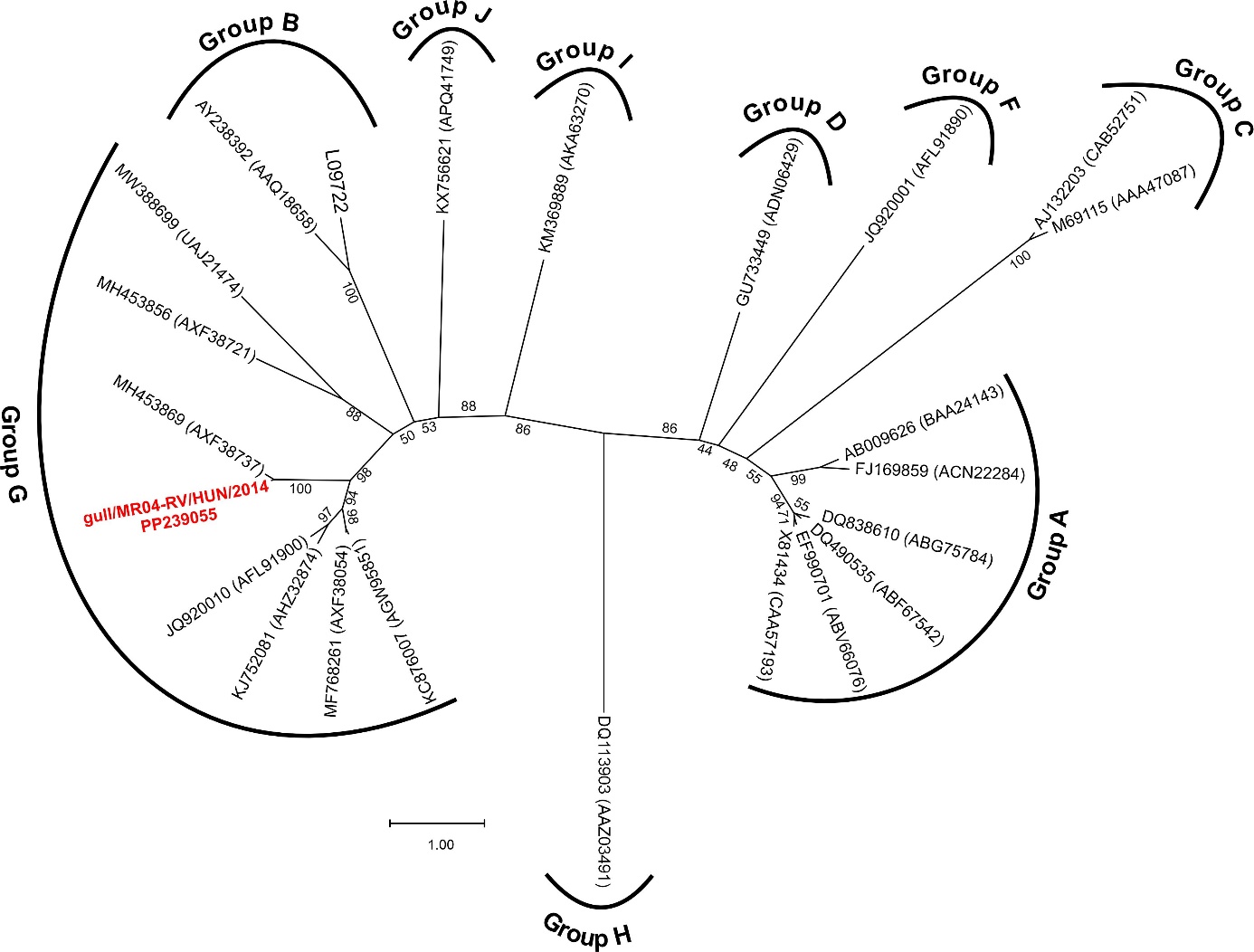


segment 10 – NSP4


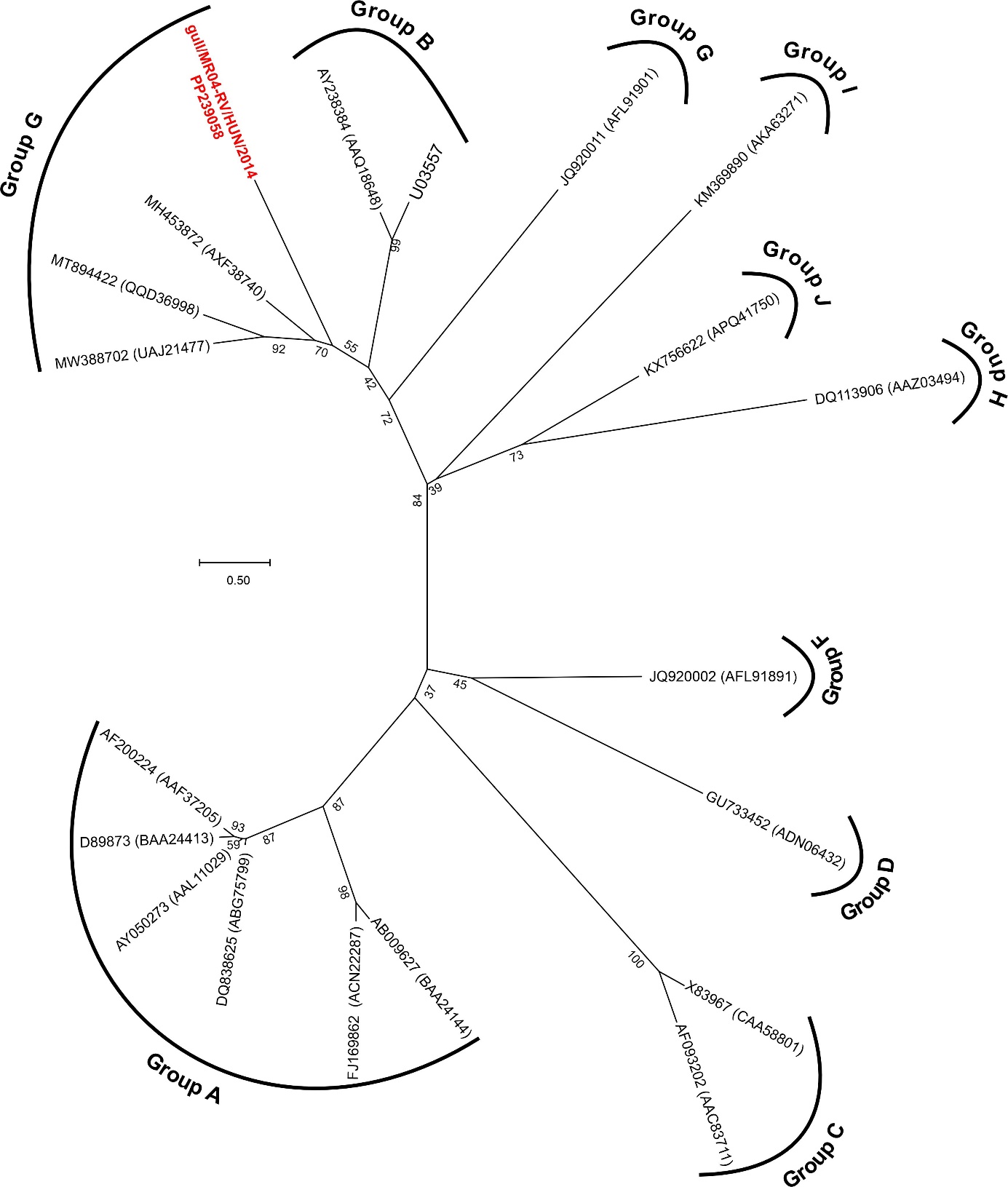


segment 11 – NSP5/NSP6


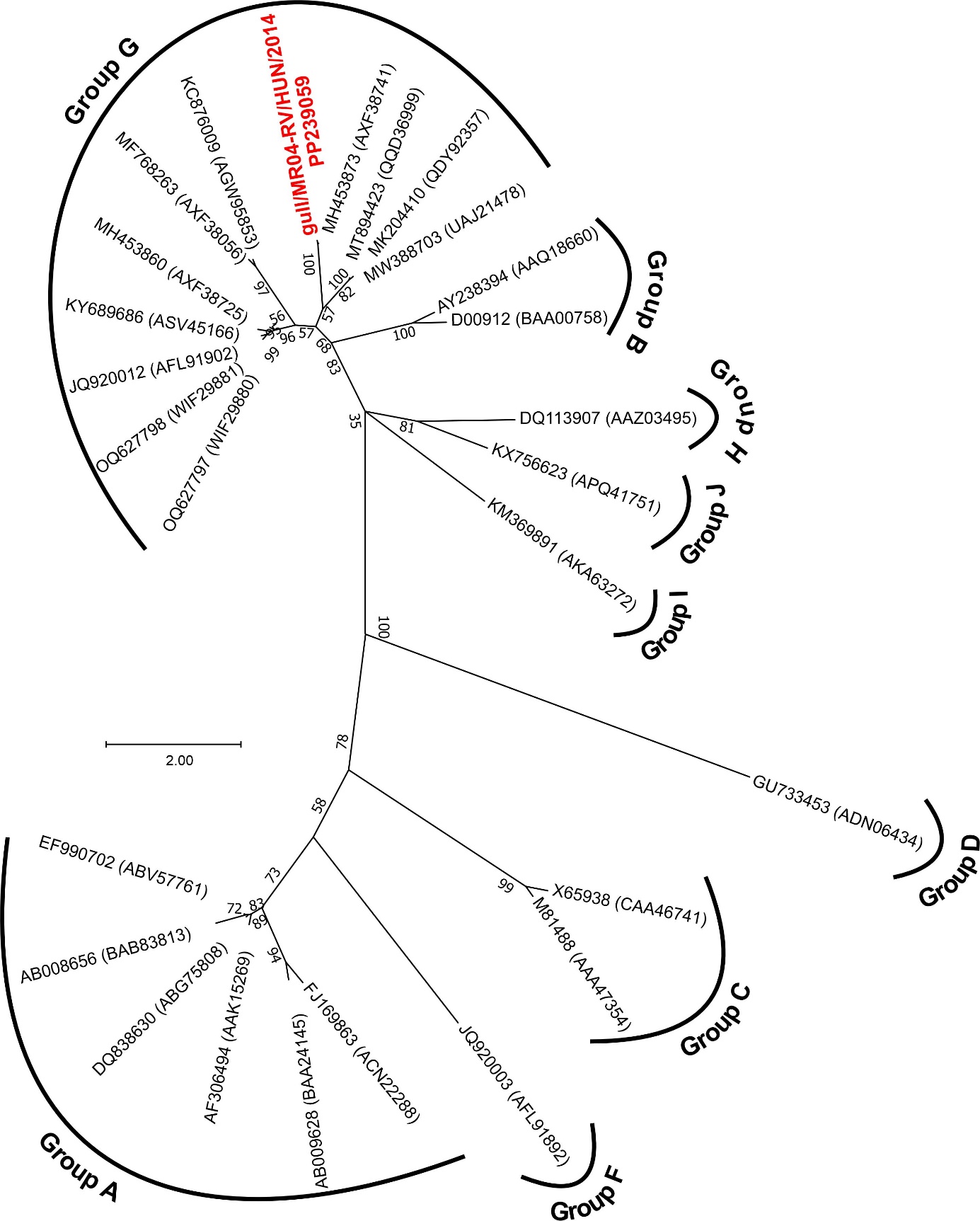


segment 2 / VP2


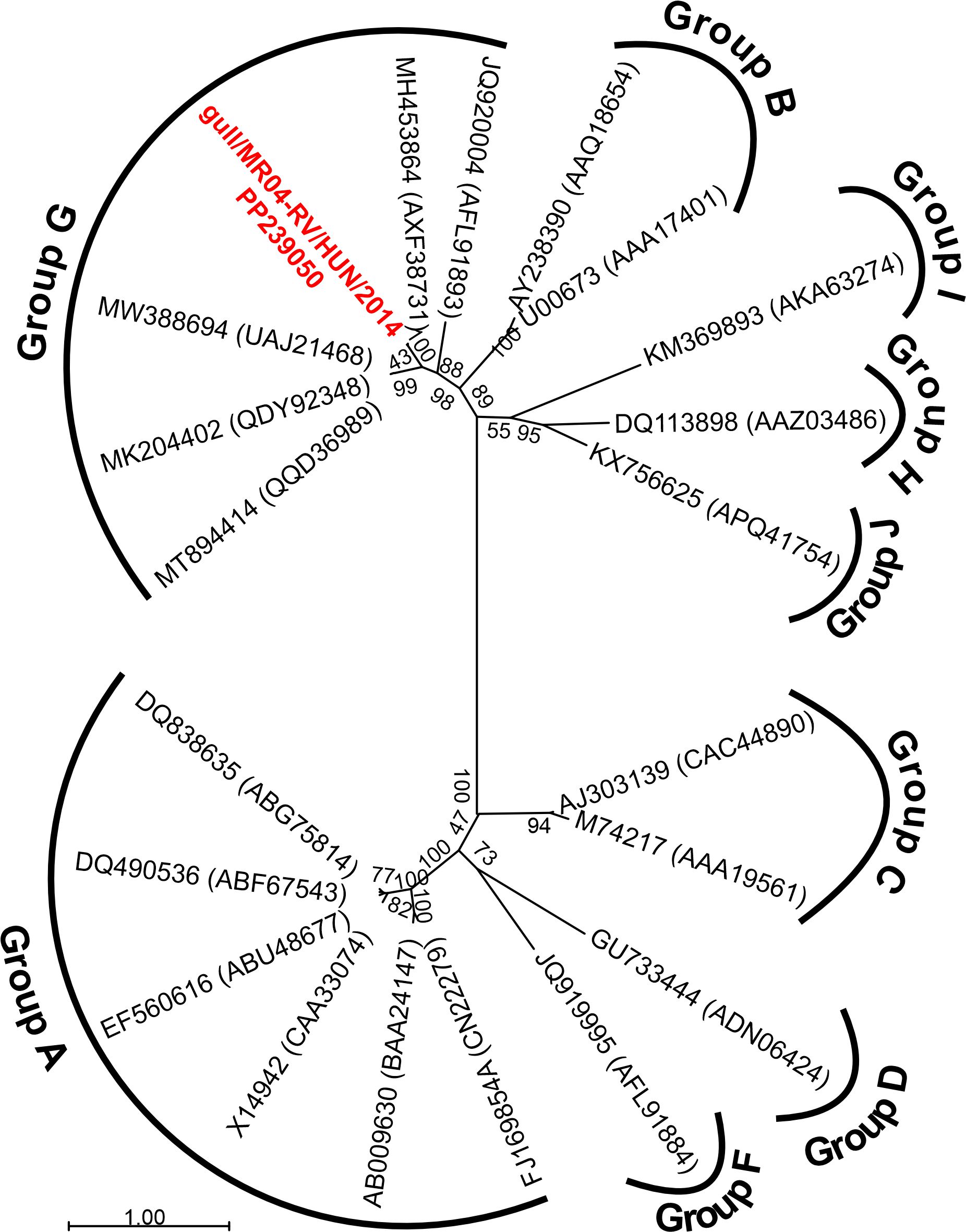


segment 6 / VP6


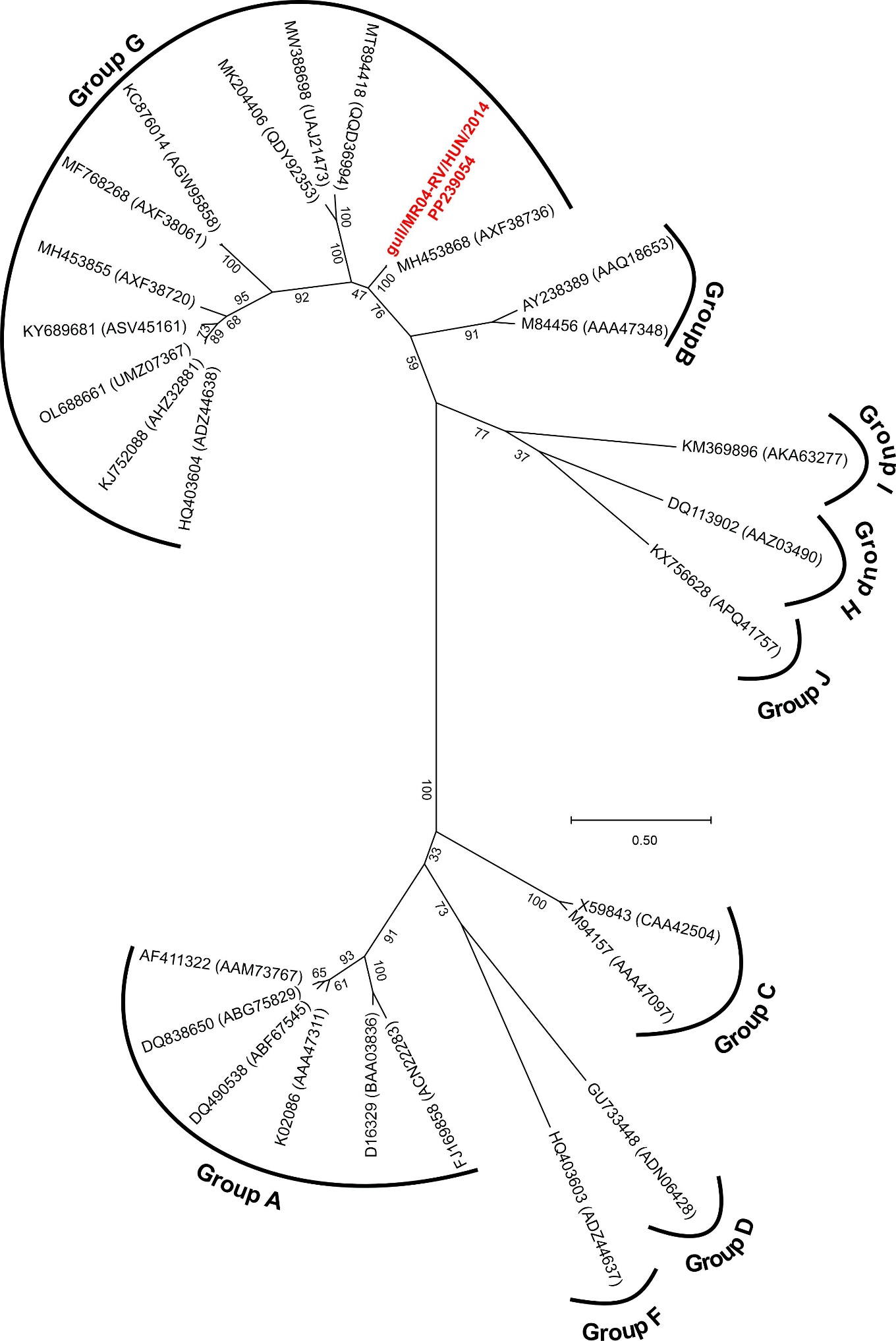


segment 9 – VP7


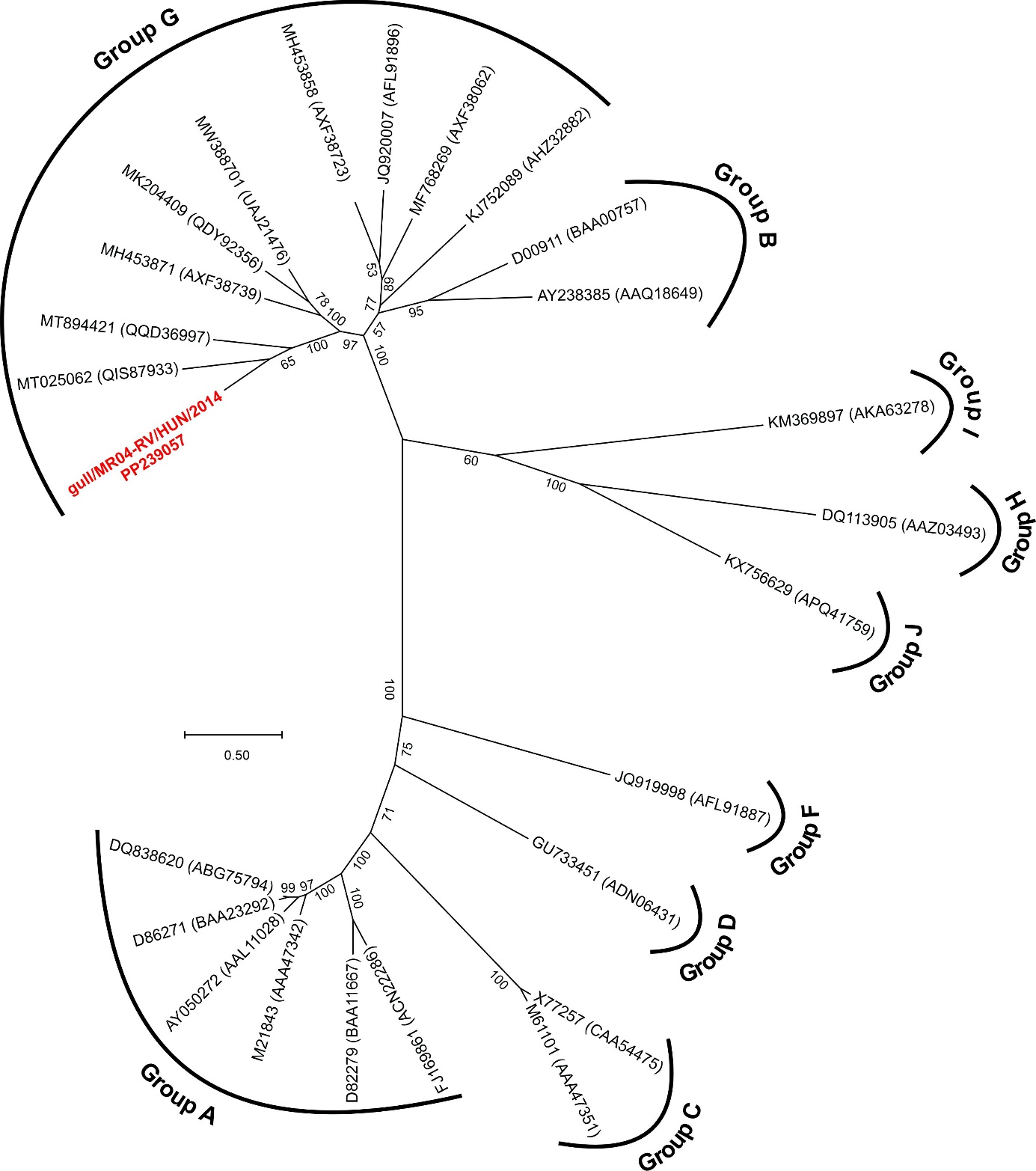


segment 4 / VP4 (VP5/VP8)


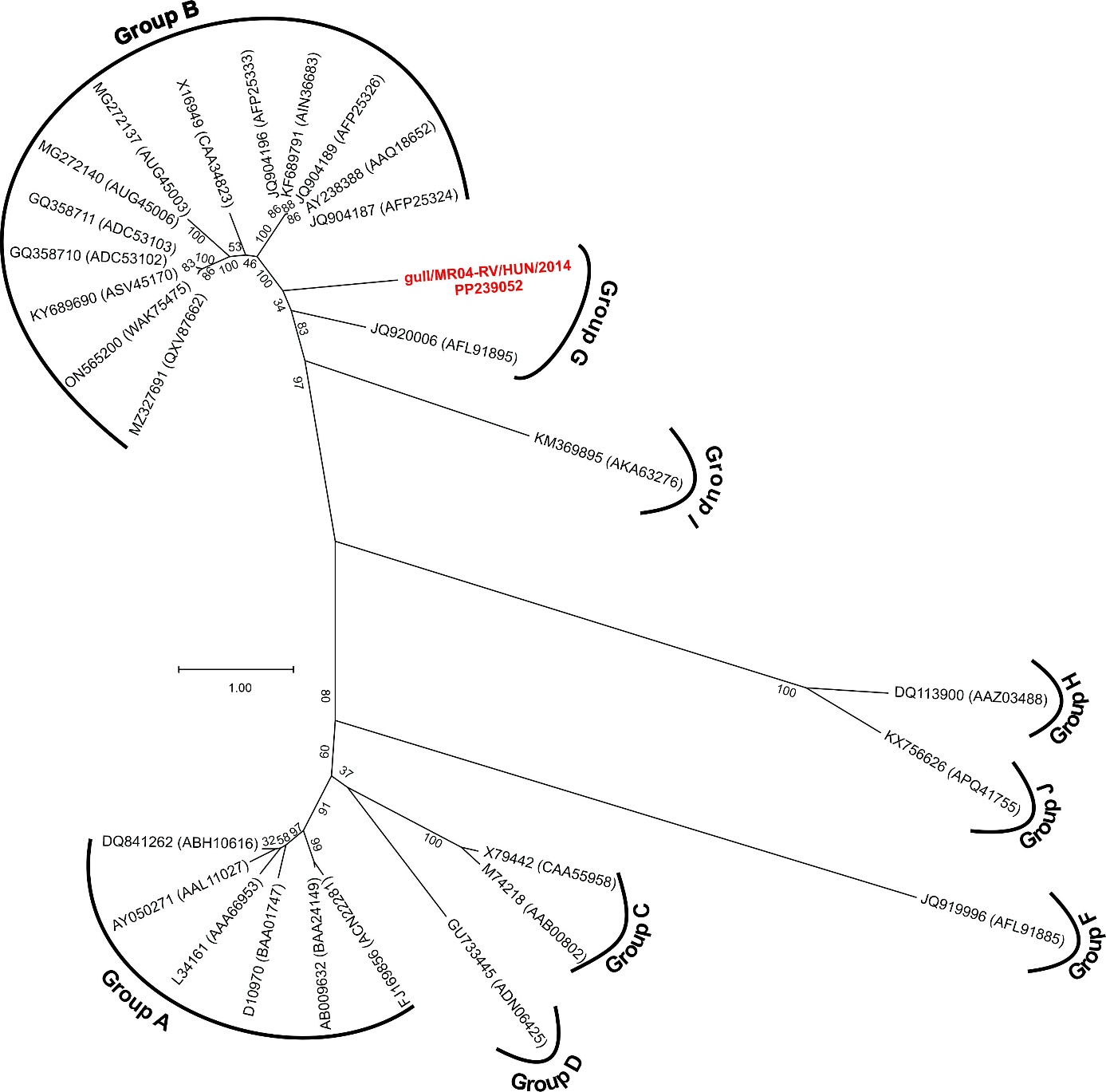


References

1. Tamura K, Stecher G, Kumar S, MEGA11: Molecular Evolutionary Genetics Analysis version 11. Mol Biol Evol. 2021; 38: 3022-3027. doi: 10.1093/molbev/msab120
