# Supplementary material for: Identification of a potential interspecies reassortant rotavirus G and avastrovirus 2 co-infection from black-headed gull (Chroicocephalus ridibundus) in Hungary
Source: PLoS One. 2025 Mar 24;20(3):e0317400. doi: 10.1371/journal.pone.0317400 (PMC11932466; doi:10.1371/journal.pone.0317400)
Supplement: S4 Fig — (DOCX) [file pone.0317400.s004.docx]

**S4 Fig. Phylogenetic and Cluster analysis of avastroviruses.**

A total of 821 avastrovirus capsid sequences were collected for analysis. **A)** The unrooted phylogenetic tree was carried out after ClustalW alignment of sequences and Maximum Likelihood statistical method, Lg with freqs (+F) model. Based on the complete amino acid sequences of the capsid protein (ORF2), avastroviruses divided into three (officially) groups: avian astrovirus 1 (AAstV-1), avian astrovirus 2 (AAstV-2) and avian astrovirus 3 (AAstV-3). Several avian astroviruses await systematic (re)classification, the group names of which have been marked with a question mark on the figure. **B)** The rooted phylogenetic analysis of the capsid proteins of avian nephritis viruses (ANVs) was carried out using Multiple Alignment using Fast Fourier Transform (MAFFT) alignment [1-4] and MEGA11 software [5]. The multiple sequence alignment was tested with the best DNA/protein model search (ML: Maximum likelihood method) and the most fit model: Maximum Likelihood statistical method with Lg with Freqs (+F) model was used to estimate phylogenetic analysis. Rates among sites were calculated with Gamma Distributed with invariant sites (G+I) option and bootstrap (1000 replicates) was also used. The potential groups and mean genetic distances were calculated by Kariithi et al. [6]. The sequences marked with a dashed line were further analysed. **C)** The cluster analysis of the group 6 of avian nephritis viruses (ANVs) using MEGA11 and Cluster Analysis method (written in R). The group 6 ANVs are theoretically sub-divided to group 6 A-D based on the phylogenetic analysis (**B**) and the mean group distances within groups were calculated with MEGA11 and signed as a group center.


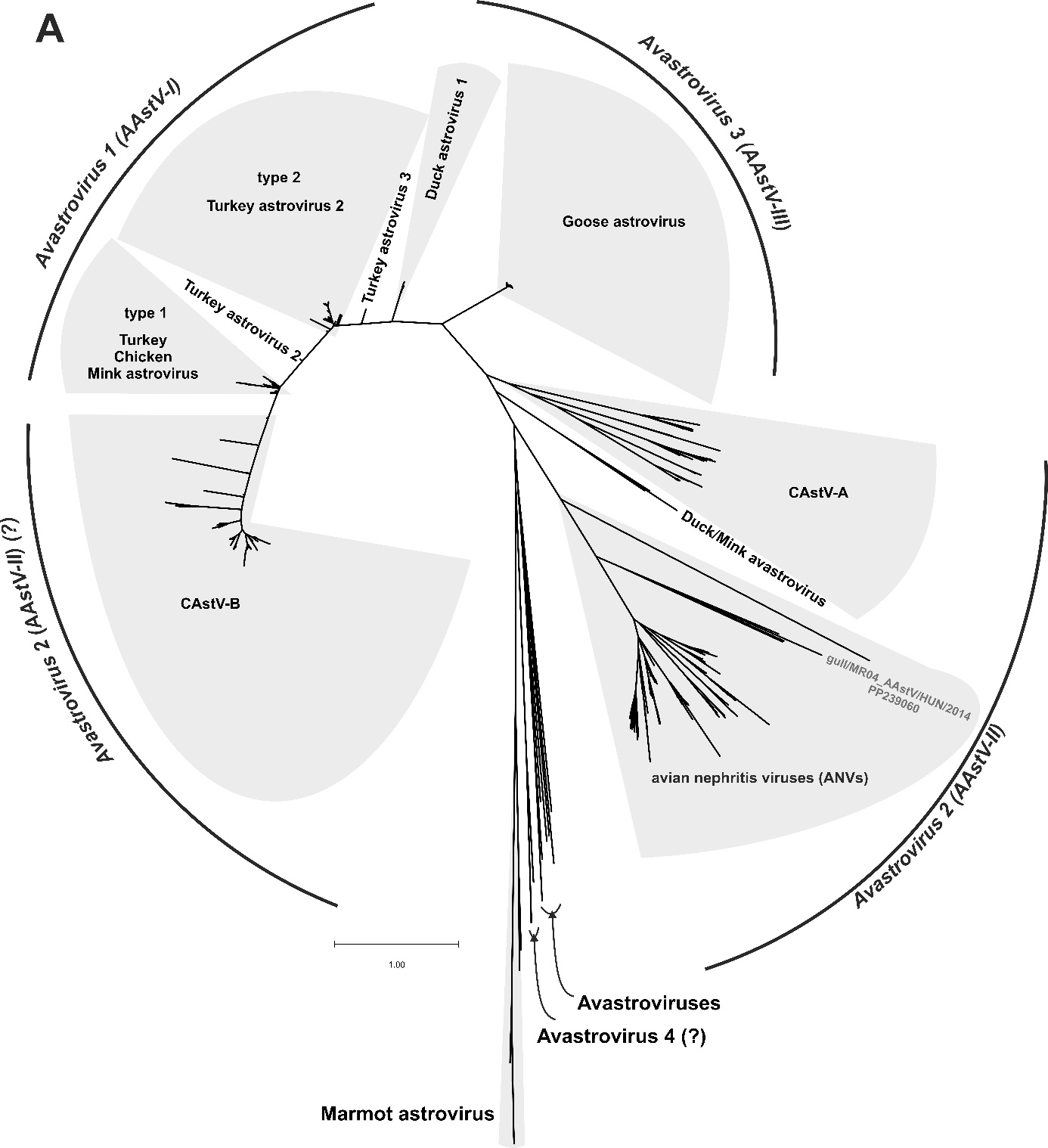


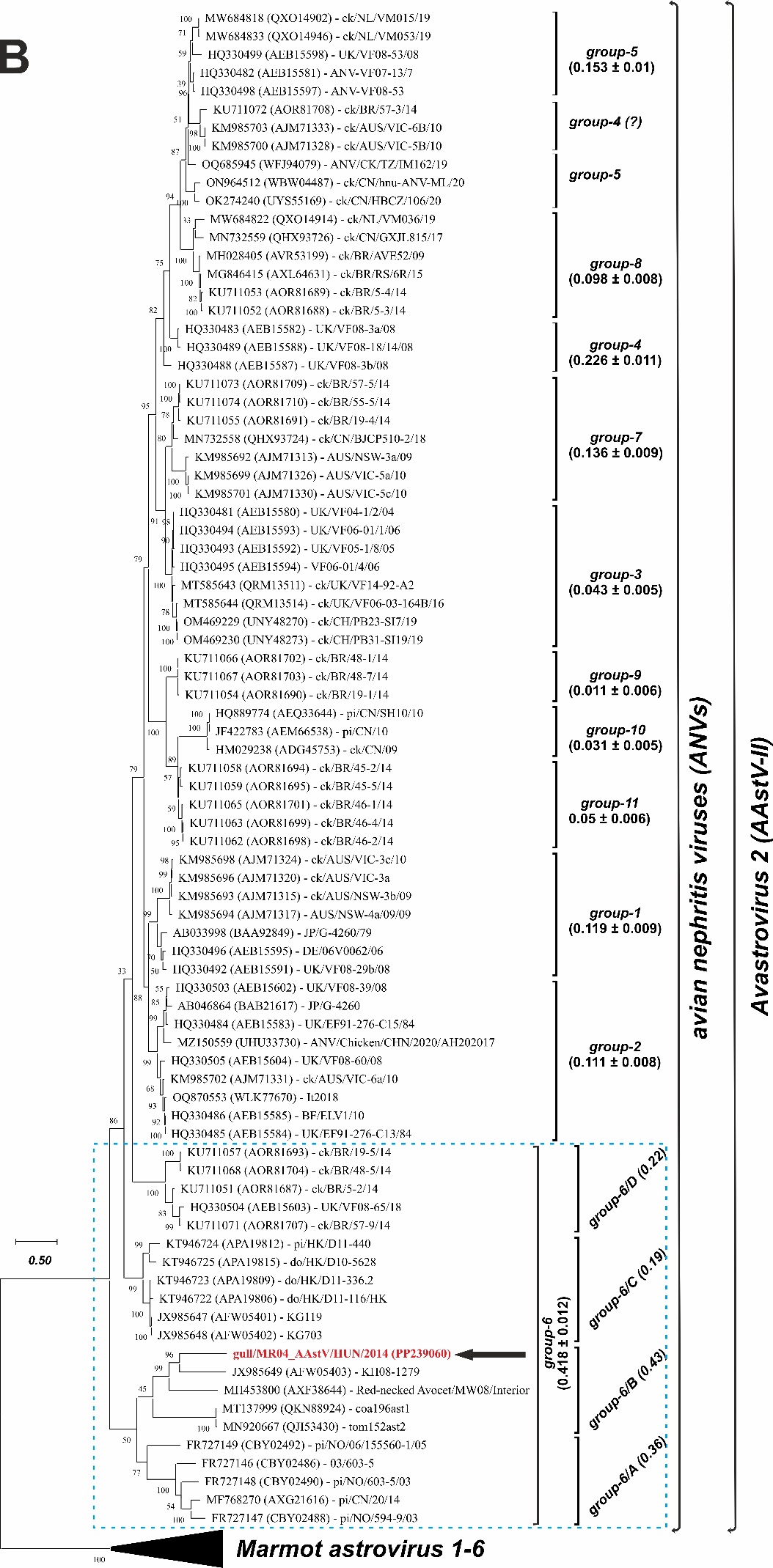


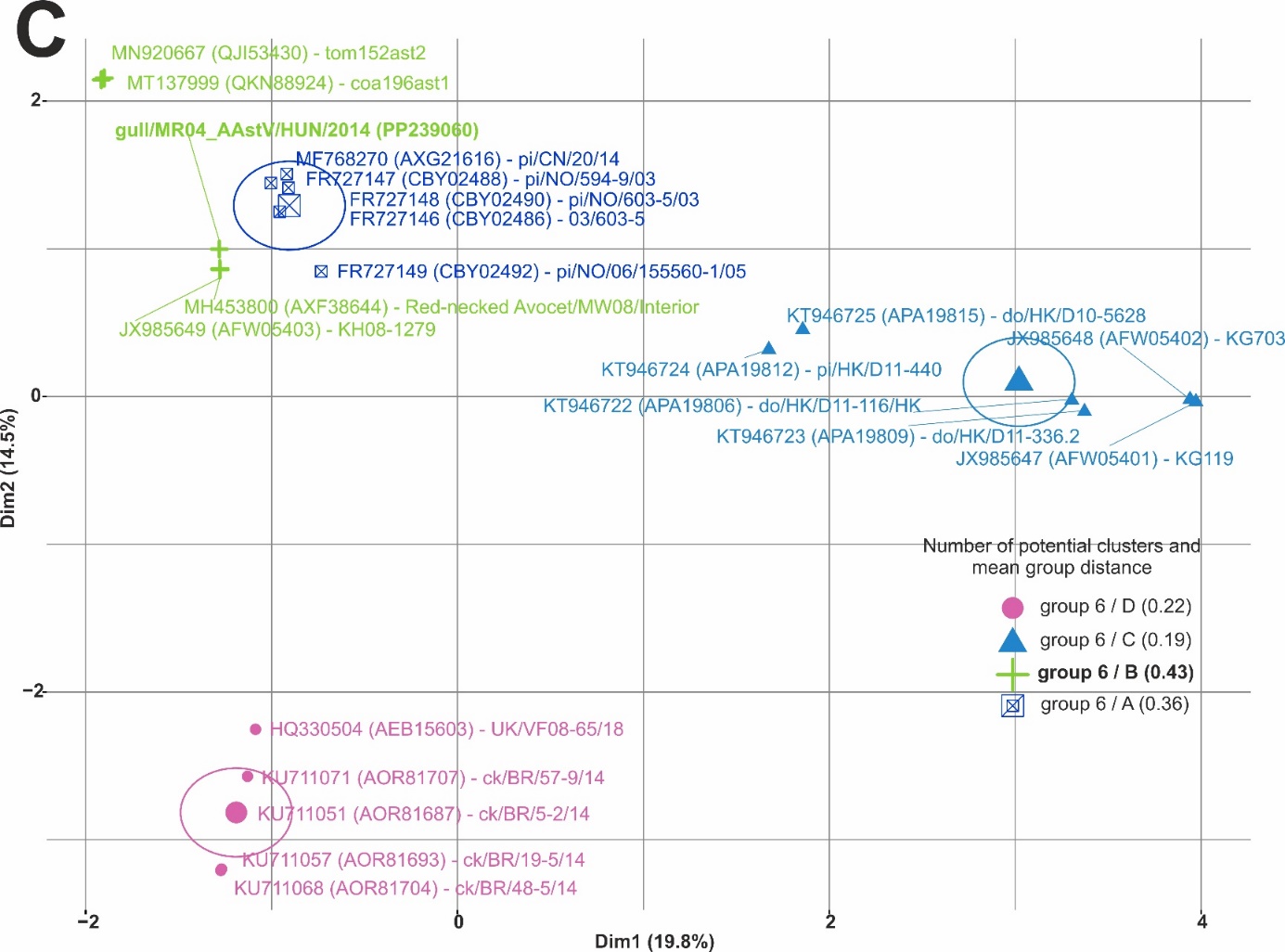


References

1. Katoh K, Rozewicki J, Yamada KD, MAFFT online service: multiple sequence alignment, interactive sequence choice and visualization. Brief Bioinform. 2019; 20: 1160-1166. doi: 10.1093/bib/bbx108
2. Minh BQ, Schmidt HA, Chernomor O, Schrempf D, Woodhams MD, von Haeseler A, et al. IQ-TREE 2: New Models and Efficient Methods for Phylogenetic Inference in the Genomic Era. Mol Biol Evol. 2020; 37: 1530-1534. doi: 10.1093/molbev/msaa015. Erratum in: Mol Biol Evol. 2020; 37: 2461.
3. Kalyaanamoorthy S, Minh BQ, Wong TKF, von Haeseler A, Jermiin LS. ModelFinder: fast model selection for accurate phylogenetic estimates. Nat Methods. 2017; 14: 587-589. doi: 10.1038/nmeth.4285
4. Hoang DT, Chernomor O, von Haeseler A, Minh BQ, Vinh LS. UFBoot2: Improving the Ultrafast Bootstrap Approximation. Mol Biol Evol. 2018; 35: 518-522. doi: 10.1093/molbev/msx281
5. Tamura K, Stecher G, Kumar S, MEGA11: Molecular Evolutionary Genetics Analysis version 11. Mol Biol Evol. 2021; 38: 3022-3027. doi: 10.1093/molbev/msab120
6. Kariithi HM, Volkening JD, Chiwanga GH, Pantin-Jackwood MJ, Msoffe PLM, Suarez DL, Genome Sequences and Characterization of Chicken Astrovirus and Avian Nephritis Virus from Tanzanian Live Bird Markets. Viruses. 2023; 15: 1247. doi: 10.3390/v15061247
