# Supplementary material for: Identification of a potential interspecies reassortant rotavirus G and avastrovirus 2 co-infection from black-headed gull (Chroicocephalus ridibundus) in Hungary
Source: PLoS One. 2025 Mar 24;20(3):e0317400. doi: 10.1371/journal.pone.0317400 (PMC11932466; doi:10.1371/journal.pone.0317400)
Supplement: S1 Table — (DOCX) [file pone.0317400.s005.docx]

**S1 Table. The genome organization of group G rotavirus strain gull/MR04-RV/HUN/2014.**

| **Supplementary Table 1**: The genome organization of group G rotavirus strain gull/MR04-RV/HUN/2014. | | | | | | |
| --- | --- | --- | --- | --- | --- | --- |
| Nucleotide accession | Protein accession | Genome segment | Segment size (nt) | Encoded protein | ORF position (nt) |  |
| PP239049 | WYV95874 | segment 1 | 3525 | VP1 (pol) (1169) | 9-3494 |  |
| PP239050 | WYV95875 | segment 2 | 3000 | VP2 (T1) (983) | 18-2969 |  |
| PP239051 | WYV95876 | segment 3 | 2343 | VP3 (cap) (763) | 9-2300 |  |
| PP239052 | WYV95877 | segment 4 | 2307 | VP4 (751) | 22-2277 |  |
| PP239053 | WYV95878 | segment 5 | 1280 | NSP1-1 (116) | 44-394 |  |
|  | WYV95879 |  |  | NSP1-2 (321) | 255-1220 |  |
| PP239054 | WYV95880 | segment 6 | 1271 | VP6 (T13) (393) | 30-1211 |  |
| PP239055 | WYV95881 | segment 7 | 1023 | NSP3 (298) | 50-946 |  |
| PP239056 | WYV95882 | segment 8 | 1014 | NSP2 (ViP) (302) | 58-966 |  |
| PP239057 | WYV95883 | segment 9 | 927 | VP7 (263) | 79-870 |  |
| PP239058 | WYV95884 | segment 10 | 936 | NSP4 (218) | 209-865 |  |
| PP239059 | WYV95885 | segment 11 | 725 | NSP5 (184) | 59-613 |  |
