# Supplementary material for: Identification of a potential interspecies reassortant rotavirus G and avastrovirus 2 co-infection from black-headed gull (Chroicocephalus ridibundus) in Hungary
Source: PLoS One. 2025 Mar 24;20(3):e0317400. doi: 10.1371/journal.pone.0317400 (PMC11932466; doi:10.1371/journal.pone.0317400)
Supplement: S2 Table — (DOCX) [file pone.0317400.s006.docx]

**S2 Table. Nucleotide and amino acid identity comparison of strain gull/MR04-RV/HUN/2014.**

Comparison of the nucleotide and the putative amino acid sequences of the study strain gull/MR04-RV/HUN/2014 and the closest relative rotavirus species corresponding segments based on NCBI blastn/blastp suite using clustered experimental database. The percent identity values with query coverage score is shown and segments with low identity values are marked in bold.

| **Study strain (name – accession)** | % nt (query cover %) | Reference (name -accession) | % aa (query cover %) | Reference (name -accession) | Rotavirus group |
| --- | --- | --- | --- | --- | --- |
| gull/MR04-RV/HUN/2014 (SEGMENT 1, PP239049) | 86% (99%) | Ruddy turnstone rotavirus isolate MW05 (MH453863) | 93.34% (99%) | viral structural protein 1 [Ruddy turnstone rotavirus] (AXF38730) | Rotavirus G |
| gull/MR04-RV/HUN/2014 (SEGMENT 2, PP239050) | 91.47 % (99%) | Ruddy turnstone rotavirus isolate MW05 (MH453864) | 98.37% (100%) | viral structural protein 2 core protein [Ruddy turnstone rotavirus] (AXF38731) | Rotavirus G |
| gull/MR04-RV/HUN/2014 (SEGMENT 3, PP239051) | 91.08% (99%) | Ruddy turnstone rotavirus isolate MW05 (MH453866) | 96.33% (100%) | viral structural protein 3 [Ruddy turnstone rotavirus] (AXF38733) | Rotavirus G |
| **gull/MR04-RV/HUN/2014 (SEGMENT 4, PP239052)** | **67.63% (41%)** | **Rotavirus B strain JN311 (KU562898)** | **40.54% (99%)** | **outer capsid spike protein VP4 [Rotavirus B] (ASV45170)** | **Rotavirus B** |
| **gull/MR04-RV/HUN/2014 (SEGMENT 5, PP239053)** | **no significant similarity found** | **-** | **35.24% (96%)** | **non-structural protein 1-2 [Rotavirus G] (ASV45160)** | **Rotavirus G** |
| gull/MR04-RV/HUN/2014 (SEGMENT 6, PP239054) | 93.33% (97%) | Ruddy turnstone rotavirus isolate MW05 (MH453868) | 99.24% (100%) | viral structural protein 6 [Ruddy turnstone rotavirus] (AXF38736) | Rotavirus G |
| gull/MR04-RV/HUN/2014 (SEGMENT 7, PP239055) | 80% (97%) | Ruddy turnstone rotavirus isolate MW05 (MH453869) | 87.25% (100%) | non-structural protein 3 [Ruddy turnstone rotavirus] (AXF38737) | Rotavirus G |
| gull/MR04-RV/HUN/2014 (SEGMENT 8, PP239056) | 94.35% (97%) | Ruddy turnstone rotavirus isolate MW05 (MH453870) | 100% (100%) | non-structural protein 2 [Ruddy turnstone rotavirus] (AXF38738) | Rotavirus G |
| **gull/MR04-RV/HUN/2014 (SEGMENT 9,** PP239057) | **71.78% (51%)** | **Shackleton virus isolate Antarctic2 (MT025062)** | **60% (88%)** | **VP7 [Pacific black duck rotavirus G] (QQD36997)** | **Rotavirus G** |
| **gull/MR04-RV/HUN/2014 (SEGMENT 10,** PP239058) | **73.74% (10%)** | **Rotavirus B** **strain RVB/Pig-wt/VNM/14176_8/NSP4 (KX362398)** | **34.36% (87%)** | **enterotoxin, partial [Rotavirus G] (****UAJ21477)** | **Rotavirus B / G** |
| gull/MR04-RV/HUN/2014 (SEGMENT 11, PP239059) | 86.76% (95%) | Ruddy turnstone rotavirus isolate MW05 (MH453873) | 95.11% (100%) | non-structural protein 5 [Ruddy turnstone rotavirus] (AXF38741) | Rotavirus G |
