# Supplementary material for: Identification of a potential interspecies reassortant rotavirus G and avastrovirus 2 co-infection from black-headed gull (Chroicocephalus ridibundus) in Hungary
Source: PLoS One. 2025 Mar 24;20(3):e0317400. doi: 10.1371/journal.pone.0317400 (PMC11932466; doi:10.1371/journal.pone.0317400)
Supplement: S3 Table — (DOCX) [file pone.0317400.s007.docx]

**S3 Table. List of rotavirus sequences included in the analysis.**

The sequences included in the table (nucleic acid/amino acid) were used for cluster and phylogenetic analysis and to detect conserved amino acid hotspots.

| **Accession number** | **Protein id** | **Organism / Strain** | **Segment / Gene** | **Host species** | **Rotavirus group** |
| --- | --- | --- | --- | --- | --- |
| AB009629 | BAA24146 | Avian rotavirus PO-13 | segment 1 / VP1 | Pigeon | Rotavirus A |
| DQ490533 | ABF67540 | RVA/Human-tc/JPN/AU-1/1982/G3P3[9] | segment 1 / VP1 | Human | Rotavirus A |
| DQ490539 | ABF67546 | RVA/Human-tc/USA/Wa/1974/G1P1A[8] | segment 1 / VP1 | Human | Rotavirus A |
| DQ838640 | ABG75819 | RVA/Simian-tc/ZAF/SA11-H96/1958/G3P5B[2] | segment 1 / VP1 | Simian | Rotavirus A |
| FJ169853 | ACN22278 | 02V0002G3 | segment 1 / VP1 | Chicken | Rotavirus A |
| EU490415 | ACD39821 | Bang373 | segment 1 / VP1 | Human | Rotavirus B |
| M97203 | P35942 | IDIR | segment 1 / VP1 | Rat | Rotavirus B |
| AJ304859 | CAC44891 | Bristol | segment 1 / VP1 | Human | Rotavirus C |
| M74216 | P26190 | Cowden | segment 1 / VP1 | Porcine | Rotavirus C |
| GU733443 | ADN06423 | Rotavirus D chicken/05V0049/DEU/2005 | segment 1 / VP1 | Chicken | Rotavirus D |
| JN596591 | AFK65656 | Rotavirus F chicken/03V0568/DEU/2003 | segment 1 / VP1 | Chicken | Rotavirus F |
| JN596592 | AFK65657 | Rotavirus G chicken/03V0567/DEU/2003 | segment 1 / VP1 | Chicken | Rotavirus G |
| DQ113897 | AAZ03485 | Adult diarrheal rotavirus strain J19 | segment 1 / VP1 | Human | Rotavirus H |
| KM369892 | AKA63273 | KE135/2012 | segment 1 / VP1 | Canine | Rotavirus I |
| KX756624 | APQ41753 | BO4351/Ms/2014 | segment 1 / VP1 | Bat | Rotavirus J |
|  |  |  |  |  |  |
| AB009630 | BAA24147 | Avian rotavirus PO-13 | segment 2 / VP2 | Pigeon | Rotavirus A |
| DQ490536 | ABF67543 | RVA/Human-tc/JPN/AU-1/1982/G3P3[9] | segment 2 / VP2 | Human | Rotavirus A |
| DQ838635 | ABG75814. | RVA/Simian-tc/ZAF/SA11-H96/1958/G3P5B[2] | segment 2 / VP2 | Simian | Rotavirus A |
| EF560616 | ABU48677 | RVA/Cow-tc/USA/WC3/1981/G6P[5] | segment 2 / VP2 | Bovine | Rotavirus A |
| FJ169854 | ACN22279 | 02V0002G3 | segment 2 / VP2 | Chicken | Rotavirus A |
| X14942 | CAA33074 | Wa | segment 2 / VP2 | Bovine | Rotavirus A |
| AY238390 | AAQ18654 | Bang373 | segment 2 / VP2 | Human | Rotavirus B |
| U00673 | AAA17401 | IDIR agent (Infectious Diarrhea of Infant Rats) | segment 2 / VP2 | Rat | Rotavirus B |
| AJ303139 | CAC44890 | Bristol | segment 2 / VP2 | Human | Rotavirus C |
| M74217 | AAA19561 | Porcine rotavirus C | segment 2 / VP2 | Porcine | Rotavirus C |
| GU733444 | ADN06424 | chicken/05V0049/DEU/2005 | segment 2 / VP2 | Chicken | Rotavirus D |
| JQ919995 | AFL91884 | chicken/03V0568/DEU/2003 | segment 2 / VP2 | Chicken | Rotavirus F |
| JQ920004 | AFL91893 | chicken/03V0567/DEU/2003 | segment 2 / VP2 | Chicken | Rotavirus G |
| DQ113898 | AAZ03486 | Adult diarrheal rotavirus strain J19 | segment 2 / VP2 | Human | Rotavirus H |
| KM369893 | AKA63274 | KE135/2012 | segment 2 / VP2 | Canine | Rotavirus I |
| KX756625 | APQ41754 | BO4351/Ms/2014 | segment 2 / VP2 | Bat | Rotavirus J |
|  |  |  |  |  |  |
| AB009631 | BAA24148 | Avian rotavirus PO-13 | segment 3 / VP3 | Pigeon | Rotavirus A |
| AY267335 | AAQ02692 | Wa | segment 3 / VP3 | Human | Rotavirus A |
| DQ490537 | ABF67544 | RVA/Human-tc/JPN/AU-1/1982/G3P3[9] | segment 3 / VP3 | Human | Rotavirus A |
| DQ838645 | ABG75824 | RVA/Simian-tc/ZAF/SA11-H96/1958/G3P5B[2] | segment 3 / VP3 | Simian | Rotavirus A |
| EF560617 | ABU48678 | RVA/Cow-tc/USA/WC3/1981/G6P[5] | segment 3 / VP3 | Bovine | Rotavirus A |
| FJ169855 | ACN22280 | 02V0002G3 | segment 3 / VP3 | Chicken | Rotavirus A |
| EU490418 | ACD39824 | Bang373 | segment 3 / VP3 | Human | Rotavirus B |
| U03556 | - | IDIR | segment 3 / VP3 | Rat | Rotavirus B |
| M74219 | AAA99239 | Porcine rotavirus C | segment 3 / VP3 | Porcine | Rotavirus C |
| X96697 | CAA65469 | Bristol | segment 3 / VP3 | Human | Rotavirus C |
| GU733446 | ADN06426 | chicken/05V0049/DEU/2005 | segment 3 / VP3 | Chicken | Rotavirus D |
| JQ919997 | AFL91886 | chicken/03V0568/DEU/2003 | segment 3 / VP3 | Chicken | Rotavirus F |
| JQ920006 | AFL91895 | chicken/03V0567/DEU/2003 | segment 3 / VP3 | Chicken | Rotavirus G |
| DQ113899 | AAZ03487 | Adult diarrheal rotavirus strain J19 | segment 3 / VP3 | Human | Rotavirus H |
| KM369894 | AKA63275 | KE135/2012 | segment 3 / VP3 | Canine | Rotavirus I |
| KX756627 | APQ41756 | BO4351/Ms/2014 | segment 3 / VP3 | Bat | Rotavirus J |
|  |  |  |  |  |  |
| AY050271 | AAL11027 | WC3 | segment 4 / VP4 | Bovine | Rotavirus A |
| D10970 | BAA01747 | Human rotavirus AU-1 | segment 4 / VP4 | Human | Rotavirus A |
| AB009632 | BAA24149 | Avian rotavirus PO-13 | segment 4 / VP4 | Pigeon | Rotavirus A |
| DQ841262 | ABH10616 | RVA/Simian-tc/ZAF/SA11-H96/1958/G3P5B[2] | segment 4 / VP4 | Simian | Rotavirus A |
| FJ169856 | ACN22281 | 02V0002G3 | segment 4 / VP4 | Chicken | Rotavirus A |
| L34161 | AAA66953 | Wa | segment 4 / VP4 | Human | Rotavirus A |
| AY238388 | AAQ18652 | Bang373 | segment 4 / VP4 | Human | Rotavirus B |
| X16949 | CAA34823 | IDIR agent | segment 4 / VP4 | Rat | Rotavirus B |
| M74218 | AAB00802 | Porcine rotavirus C | segment 4 / VP4 | Porcine | Rotavirus C |
| X79442 | CAA55958 | Bristol | segment 4 / VP4 | Human | Rotavirus C |
| GU733445 | ADN06425 | chicken/05V0049/DEU/2005 | segment 4 / VP4 | Chicken | Rotavirus D |
| JQ919996 | AFL91885 | chicken/03V0568/DEU/2003 | segment 4 / VP4 | Chicken | Rotavirus F |
| JQ920006 | AFL91895 | chicken/03V0567/DEU/2003 | segment 4 / VP4 | Chicken | Rotavirus G |
| DQ113900 | AAZ03488 | Adult diarrheal rotavirus strain J19 | segment 4 / VP4 | Human | Rotavirus H |
| KM369895 | AKA63276 | KE135/2012 | segment 4 / VP4 | Canine | Rotavirus I |
| KX756626 | APQ41755 | BO4351/Ms/2014 | segment 4 / VP4 | Bat | Rotavirus J |
|  |  |  |  |  |  |
| D16329 | BAA03836 | Avian rotavirus PO-13 | segment 6 / VP6 | Pigeon | Rotavirus A |
| AF411322 | AAM73767 | WC3 | segment 6 / VP6 | Bovine | Rotavirus A |
| DQ490538 | ABF67545 | RVA/Human-tc/JPN/AU-1/1982/G3P3[9] | segment 6 / VP6 | Human | Rotavirus A |
| DQ838650 | ABG75829 | RVA/Simian-tc/ZAF/SA11-H96/1958/G3P5B[2] | segment 6 / VP6 | Simian | Rotavirus A |
| FJ169858 | ACN22283 | 02V0002G3 | segment 6 / VP6 | Chicken | Rotavirus A |
| K02086 | AAA47311 | Wa | segment 6 / VP6 | Human | Rotavirus A |
| AY238389 | AAQ18653 | Bang373 | segment 6 / VP6 | Human | Rotavirus B |
| M84456 | AAA47348 | IDIR | segment 6 / VP6 | Rat | Rotavirus B |
| M94157 | AAA47097 | Cowden | segment 6 / VP6 | Porcine | Rotavirus C |
| X59843 | CAA42504 | Bristol | segment 6 / VP6 | Human | Rotavirus C |
| GU733448 | ADN06428 | chicken/05V0049/DEU/2005 | segment 6 / VP6 | Chicken | Rotavirus D |
| HQ403603 | ADZ44637 | chicken/03V0568/DEU/2003 | segment 6 / VP6 | Chicken | Rotavirus F |
| DQ113902 | AAZ03490 | Adult diarrheal rotavirus strain J19 | segment 6 / VP6 | Human | Rotavirus H |
| HQ403604 | ADZ44638 | chicken/03V0567/DEU/2003 | segment 6 / VP6 | Chicken | Roatvirus G |
| KM369896 | AKA63277 | KE135/2012 | segment 6 / VP6 | Canine | Rotavirus I |
| KX756628 | APQ41757 | BO4351/Ms/2014 | segment 6 / VP6 | Bat | Rotavirus J |
|  |  |  |  |  |  |
| AY050272 | AAL11028 | WC3 | segment 9 / VP7 | Bovine | Rotavirus A |
| D82979 | BAA11667 | Avian rotavirus PO-13 | segment 9 / VP7 | Pigeon | Rotavirus A |
| D86271 | BAA23292 | AU-1 | segment 9 / VP7 | Human | Rotavirus A |
| DQ838620 | ABG75794 | RVA/Simian-tc/ZAF/SA11-H96/1958/G3P5B[2] | segment 9 / VP7 | Simian | Rotavirus A |
| FJ169861 | ACN22286 | 02V0002G3 | segment 9 / VP7 | Chicken | Rotavirus A |
| M21843 | AAA47342 | Wa | segment 9 / VP7 | Human | Rotavirus A |
| AY238385 | AAQ18649 | Bang373 | segment 9 / VP7 | Human | Rotavirus B |
| D00911 | BAA00757 | IDIR agent | segment 9 / VP7 | Rat | Rotavirus B |
| AJ132205 | CAB52753 | Bristol | segment 9 / VP7 | Human | Rotavirus C |
| M61101 | AAA47351 | Cowden | segment 9 / VP7 | Porcine | Rotavirus C |
| X77257 | CAA54475 | Bristol | segment 9 / VP7 | Human | Rotavirus C |
| GU733451 | ADN06431 | chicken/05V0049/DEU/2005 | segment 9 / VP7 | Chicken | Rotavirus D |
| JQ919998 | AFL91887 | chicken/03V0568/DEU/2003 | segment 9 / VP7 | Chicken | Rotavirus F |
| JQ920007 | AFL91896 | chicken/03V0567/DEU/2003 | segment 9 / VP7 | Chicken | Rotavirus G |
| DQ113905 | AAZ03493 | Adult diarrheal rotavirus strain J19 | segment 9 / VP7 | Human | Rotavirus H |
| KM369897 | AKA63278 | KE135/2012 | segment 9 / VP7 | Canine | Rotavirus I |
| KX756629 | APQ41759 | BO4351/Ms/2014 | segment 9 / VP7 | Bat | Rotavirus J |
|  |  |  |  |  |  |
| AB009633 | BAA24150 | Avian rotavirus PO-13 | segment 5 / NSP1 | Pigeon | Rotavirus A |
| D45244 | BAA08200 | AU-1 | segment 5 / NSP1 | Human | Rotavirus A |
| DQ838599 | ABG75773 | RVA/Simian-tc/ZAF/SA11-H96/1958/G3P5B[2] | segment 5 / NSP1 | Simian | Rotavirus A |
| EF990699 | ABV66088 | RVA/Cow-tc/USA/WC3/1981/G6P[5] | segment 5 / NSP1 | Bovine | Rotavirus A |
| FJ169857 | ACN22282 | 02V0002G3 | segment 5 / NSP1 | Chicken | Rotavirus A |
| L18943 | AAA02910 | Wa | segment 5 / NSP1 | Human | Rotavirus A |
| U01164 | AAA60453 | IDIR agent (Infectious Diarrhea of Infant Rats) | segment 5 / NSP1 | Rat | Rotavirus B |
| AY238391 | AAQ18656 | Bang373 | segment 5 / NSP1 | Human | Rotavirus B |
| AJ132204 | CAB52752 | Bristol | segment 5 / NSP1 | Human | Rotavirus C |
| X59843 | CAA42504 | Bristol | segment 5 / NSP1 | Human | Rotavirus C |
| X60546 | CAA43036 | Cowden | segment 5 / NSP1 | Porcine | Rotavirus C |
| GU733447 | ADN06427 | chicken/05V0049/DEU/2005 | segment 5 / NSP1 | Chicken | Rotavirus D |
| JQ919999 | AFL91888 | chicken/03V0568/DEU/2003 | segment 5 / NSP1 | Chicken | Rotavirus F |
| JQ920008 | AFL91898 | chicken/03V0567/DEU/2003 | segment 5 / NSP1 | Chicken | Rotavirus G |
| DQ113901 | AAZ03489 | Adult diarrheal rotavirus strain J19 | segment 5 / NSP1 | Human | Rotavirus H |
| KM369887 | AKA63268 | KE135/2012 | segment 5 / NSP1 | Canine | Rotavirus I |
| KX756619 | APQ41747 | BO4351/Ms/2014 | segment 5 / NSP1 | Bat | Rotavirus J |
|  |  |  |  |  |  |
| AB009625 | BAA24142 | Avian rotavirus PO-13 | segment 8 / NSP2 | Pigeon | Rotavirus A |
| DQ490534 | ABF67541 | RVA/Human-tc/JPN/AU-1/1982/G3P3[9] | segment 8 / NSP2 | Human | Rotavirus A |
| DQ838615 | ABG75789 | RVA/Simian-tc/ZAF/SA11-H96/1958/G3P5B[2] | segment 8 / NSP2 | Simian | Rotavirus A |
| EF990700 | ABV66082 | RVA/Cow-tc/USA/WC3/1981/G6P[5] | segment 8 / NSP2 | Bovine | Rotavirus A |
| FJ169860 | ACN22285 | 02V0002G3 | segment 8 / NSP2 | Chicken | Rotavirus A |
| L04534 | AAA47301 | Wa | segment 8 / NSP2 | Human | Rotavirus A |
| AY238393 | AAQ18659 | Bang373 | segment 8 / NSP2 | Human | Rotavirus B |
| U03558 | - | IDIR agent | segment 8 / NSP2 | Rat | Rotavirus B |
| X65939 | CAA46742 | Cowden | segment 8 / NSP2 | Porcine | Rotavirus C |
| AJ132205 | CAB52753 | Bristol | segment 8 / NSP2 | Human | Rotavirus C |
| X77257 | CAA54475 | Bristol | segment 8 / NSP2 | Human | Rotavirus C |
| GU733450 | ADN06430 | chicken/05V0049/DEU/2005 | segment 8 / NSP2 | Chicken | Rotavirus D |
| JQ920000 | AFL91889 | chicken/03V0568/DEU/2003 | segment 8 / NSP2 | Chicken | Rotavirus F |
| JQ920009 | AFL91899 | chicken/03V0567/DEU/2003 | segment 8 / NSP2 | Chicken | Rotavirus G |
| DQ113904 | AAZ03492 | Adult diarrheal rotavirus strain J19 | segment 8 / NSP2 | Human | Rotavirus H |
| KM369888 | AKA63269 | KE135/2012 | segment 8 / NSP2 | Canine | Rotavirus I |
| KX756620 | APQ41748 | BO4351/Ms/2014 | segment 8 / NSP2 | Bat | Rotavirus J |
|  |  |  |  |  |  |
| AB009626 | BAA24143 | Avian rotavirus PO-13 | segment 7 / NSP3 | Pigeon | Rotavirus A |
| DQ490535 | ABF67542 | RVA/Human-tc/JPN/AU-1/1982/G3P3[9] | segment 7 / NSP3 | Human | Rotavirus A |
| DQ838610 | ABG75784 | RVA/Simian-tc/ZAF/SA11-H96/1958/G3P5B[2] | segment 7 / NSP3 | Simian | Rotavirus A |
| EF990701 | ABV66076 | RVA/Cow-tc/USA/WC3/1981/G6P[5] | segment 7 / NSP3 | Bovine | Rotavirus A |
| FJ169859 | ACN22284 | 02V0002G3 | segment 7 / NSP3 | Chicken | Rotavirus A |
| X81434 | CAA57193 | Wa | segment 7 / NSP3 | Human | Rotavirus A |
| AY238392 | AAQ18658 | Bang373 | segment 7 / NSP3 | Human | Rotavirus B |
| L09722 | - | IDIR | segment 7 / NSP3 | Rat | Rotavirus B |
| AJ132203 | CAB52751 | Bristol | segment 7 / NSP3 | Human | Rotavirus C |
| AJ132204 | CAB52752 | Bristol | segment 7 / NSP3 | Human | Rotavirus C |
| M69115 | AAA47087 | Cowden | segment 7 / NSP3 | Porcine | Rotavirus C |
| GU733449 | ADN06429 | chicken/05V0049/DEU/2005 | segment 7 / NSP3 | Chicken | Rotavirus D |
| JQ920001 | AFL91890 | chicken/03V0568/DEU/2003 | segment 7 / NSP3 | Chicken | Rotavirus F |
| JQ920010 | AFL91900 | chicken/03V0567/DEU/2003 | segment 7 / NSP3 | Chicken | Rotavirus G |
| DQ113903 | AAZ03491 | Adult diarrheal rotavirus strain J19 | segment 7 / NSP3 | Human | Rotavirus H |
| KM369889 | AKA63270 | KE135/2012 | segment 7 / NSP3 | Canine | Rotavirus I |
| KX756621 | APQ41749 | BO4351/Ms/2014 | segment 7 / NSP3 | Bat | Rotavirus J |
|  |  |  |  |  |  |
| AB009627 | BAA24144 | Avian rotavirus PO-13 | segment 10 / NSP4 | Pigeon | Rotavirus A |
| AF200224 | AAF37205 | Wa | segment 10 / NSP4 | Human | Rotavirus A |
| AY050273 | AAL11029 | WC3 | segment 10 / NSP4 | Bovine | Rotavirus A |
| D89873 | BAA24413 | RVA/Human-tc/JPN/AU-1/1982/G3P3[9] | segment 10 / NSP4 | Human | Rotavirus A |
| DQ838625 | ABG75799 | RVA/Simian-tc/ZAF/SA11-H96/1958/G3P5B[2] | segment 10 / NSP4 | Simian | Rotavirus A |
| FJ169862 | ACN22287 | 02V0002G3 | segment 10 / NSP4 | Chicken | Rotavirus A |
| AY238384 | AAQ18648 | Bang373 | segment 10 / NSP4 | Human | Rotavirus B |
| U03557 | - | IDIR | segment 10 / NSP4 | Rat | Rotavirus B |
| AF093202 | AAC83711 | Cowden | segment 10 / NSP4 | Porcine | Rotavirus C |
| M81488 | AAA47354 | Bristol | segment 10 / NSP4 | Human | Rotavirus C |
| X83967 | CAA58801 | Bristol | segment 10 / NSP4 | Human | Rotavirus C |
| GU733452 | ADN06432 | chicken/05V0049/DEU/2005 | segment 10 / NSP4 | Chicken | Rotavirus D |
| JQ920003 | AFL91892 | chicken/03V0568/DEU/2003 | segment 10 / NSP4 | Chicken | Rotavirus F |
| JQ920011 | AFL91901 | chicken/03V0567/DEU/2003 | segment 10 / NSP4 | Chicken | Rotavirus G |
| DQ113906 | AAZ03494 | Adult diarrheal rotavirus strain J19 | segment 10 / NSP4 | Human | Rotavirus H |
| KM369890 | AKA63271 | KE135/2012 | segment 10 / NSP4 | Canince | Rotavirus I |
| KX756622 | APQ41750 | BO4351/Ms/2014 | segment 10 / NSP4 | Bat | Rotavirus J |
|  |  |  |  |  |  |
| AB008656 | BAB83813 | AU-1 | segment 11 / NSP5 | Human | Rotavirus A |
| AB009628 | BAA24145 | Avian rotavirus PO-13 | segment 11 / NSP5 | Pigeon | Rotavirus A |
| AF306494 | AAK15269 | Wa | segment 11 / NSP5 | Human | Rotavirus A |
| DQ838630 | ABG75808 | RVA/Simian-tc/ZAF/SA11-H96/1958/G3P5B[2] | segment 11 / NSP5 | Simian | Rotavirus A |
| EF990702 | ABV57761 | RVA/Cow-tc/USA/WC3/1981/G6P[5] | segment 11 / NSP5 | Bovine | Rotavirus A |
| FJ169863 | ACN22288 | 02V0002G3 | segment 11 / NSP5 | Chicken | Rotavirus A |
| AY238394 | AAQ18660 | Bang373 | segment 11 / NSP5 | Human | Rotavirus B |
| D00912 | BAA00758 | IDIR | segment 11 / NSP5 | Rat | Rotavirus B |
| M81488 | AAA47354 | Bristol | segment 11 / NSP5 | Human | Rotavirus C |
| X65938 | CAA46741 | Cowden | segment 11 / NSP5 | Porcine | Rotavirus C |
| X83967 | CAA58801 | Bristol | segment 11 / NSP5 | Human | Rotavirus C |
| GU733453 | ADN06434 | chicken/05V0049/DEU/2005 | segment 11 / NSP5 | Chicken | Rotavirus D |
| JQ920002 | AFL91891 | chicken/03V0568/DEU/2003 | segment 11 / NSP5 | Chicken | Rotavirus F |
| JQ920012 | AFL91902 | chicken/03V0567/DEU/2003 | segment 11 / NSP5 | Chicken | Rotavirus G |
| DQ113907 | AAZ03495 | Adult diarrheal rotavirus strain J19 | segment 11 / NSP5 | Human | Rotavirus H |
| KM369891 | AKA63272 | KE135/2012 | segment 11 / NSP5 | Canine | Rotavirus I |
| KX756623 | APQ41751 | BO4351/Ms/2014 | segment 11 / NSP5 | Bat | Rotavirus J |
