# Supplementary material for: Identification of a potential interspecies reassortant rotavirus G and avastrovirus 2 co-infection from black-headed gull (Chroicocephalus ridibundus) in Hungary
Source: PLoS One. 2025 Mar 24;20(3):e0317400. doi: 10.1371/journal.pone.0317400 (PMC11932466; doi:10.1371/journal.pone.0317400)
Supplement: S1 File — (DOCX) [file pone.0317400.s008.docx]

**Web references**

Magyar Madártani és Természetvédelmi Egyesület (MME), <https://mme.hu/magyarorszagmadarai/madaradatbazis-larrid> (accessed 25.03.2024)

BirdLife International, <http://datazone.birdlife.org/species/factsheet/black-headed-gull-larus-ridibundus> (accessed 25.03.2024)

International Committee on Taxonomy of Viruses: ICTV, <https://ictv.global/report/chapter/sedoreoviridae/sedoreoviridae/rotavirus> (accessed 25.03.2024)

International Committee on Taxonomy of Viruses: ICTV, <https://ictv.global/report_9th/RNApos/Astroviridae> (accessed 25.03.2024)

Integrated DNA Technologies (IDT DNA), <https://eu.idtdna.com>

National Center for Biotechnology Information, Needleman-Wunsch Global Align,

<https://blast.ncbi.nlm.nih.gov/Blast.cgi?PAGE_TYPE=BlastSearch&PROG_DEF=blastn&BLAST_PROG_DEF=blastn&BLAST_SPEC=GlobalAln&LINK_LOC=BlastHomeLink>

Multiple Sequence Alignment (MSA) – MUSCLE, <https://www.ebi.ac.uk/jdispatcher/msa/muscle>

European Centre for Disease Prevention and Control (ECDC), Disease factsheet about rotavirus. <https://www.ecdc.europa.eu/en/rotavirus-infection/facts> (accesses 10.12.2024)
